# DNA repair, recombination, and damage signaling

**DOI:** 10.1093/genetics/iyab178

**Published:** 2021-02-04

**Authors:** Anton Gartner, JoAnne Engebrecht

**Affiliations:** 1 Department for Biological Sciences, IBS Center for Genomic Integrity, Ulsan National Institute of Science and Technology, Ulsan 689-798, Republic of Korea; 2 Department of Molecular and Cellular Biology, University of California Davis, Davis, CA 95616, USA

**Keywords:** DNA repair, recombination, checkpoint signaling, WormBook

## Abstract

DNA must be accurately copied and propagated from one cell division to the next, and from one generation to the next. To ensure the faithful transmission of the genome, a plethora of distinct as well as overlapping DNA repair and recombination pathways have evolved. These pathways repair a large variety of lesions, including alterations to single nucleotides and DNA single and double-strand breaks, that are generated as a consequence of normal cellular function or by external DNA damaging agents. In addition to the proteins that mediate DNA repair, checkpoint pathways have also evolved to monitor the genome and coordinate the action of various repair pathways. Checkpoints facilitate repair by mediating a transient cell cycle arrest, or through initiation of cell suicide if DNA damage has overwhelmed repair capacity. In this chapter, we describe the attributes of *Caenorhabditis elegans* that facilitate analyses of DNA repair, recombination, and checkpoint signaling in the context of a whole animal. We review the current knowledge of *C. elegans* DNA repair, recombination, and DNA damage response pathways, and their role during development, growth, and in the germ line. We also discuss how the analysis of mutational signatures in *C. elegans* is helping to inform cancer mutational signatures in humans.

## General overview of DNA repair, recombination, and DNA damage signaling pathways 

Genome integrity is critical for normal cellular function as well as the faithful propagation of the genome through mitosis and meiosis. Multiple, partially redundant, DNA repair, recombination, and signaling pathways have evolved to counteract DNA damage that arises from both external and internal sources. These pathways are highly conserved, from bacteria to humans, although their relative use differs among species, as well as in different tissues within the same organism. Much of our understanding of DNA repair and recombination pathways has come from *in vivo* studies in *Escherichia* *coli*, yeasts, and human cell lines, in combination with *in vitro* biochemical analyses. While repair and recombination pathways are largely conserved, added complexity within pathways appears to have evolved with multicellularity and genome complexity. Current research is focused on elucidating molecular mechanisms of repair, recombination, and checkpoint signaling pathways. Additionally, understanding how these pathways are integrated and differentially regulated in development, disease, aging, within different somatic tissues, and in the germ line are important areas of investigation. *Caenorhabditis* *elegans* is an excellent system to address these outstanding questions.

**Figure 1:**
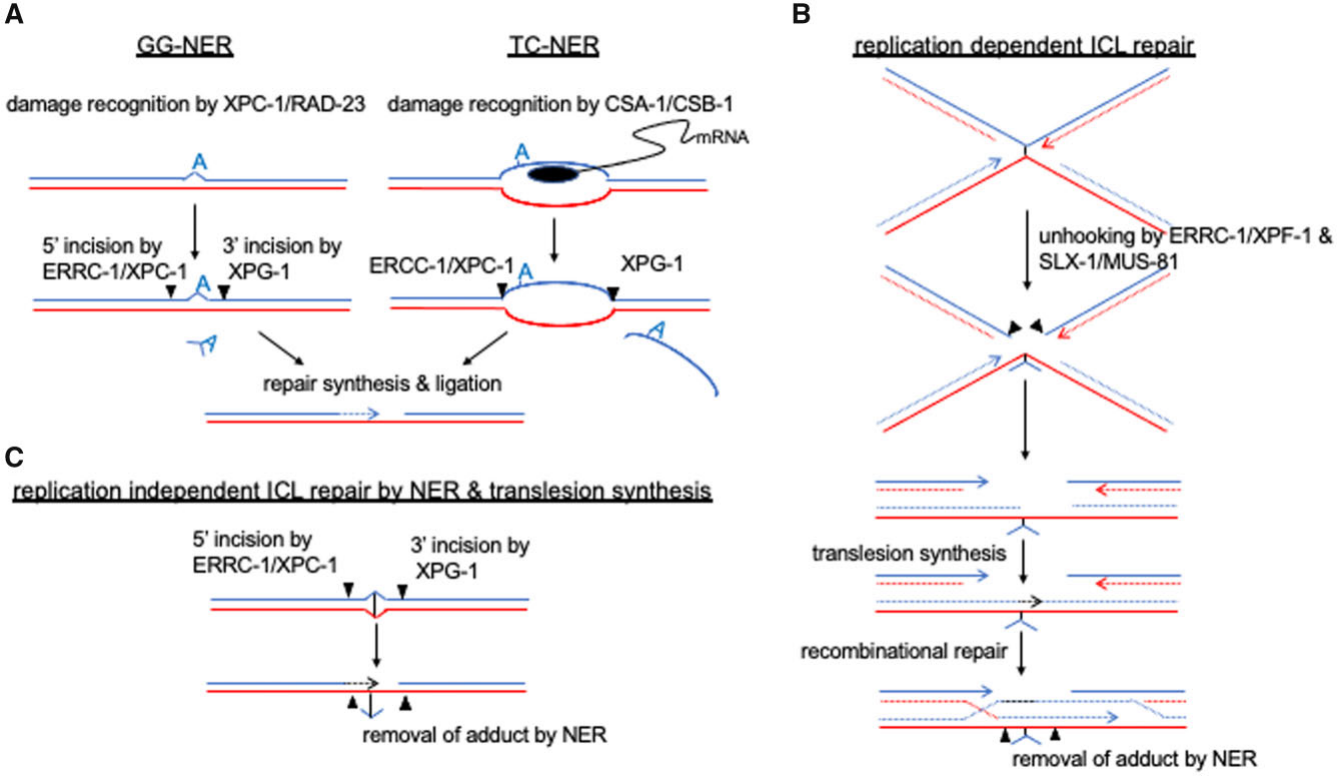
NER and replication-dependent and -independent interstrand crosslink (ICL) repair models. (A) Model for GG-NER and TC-NER (top left and middle panels). (B) Replication-dependent ICL repair and (C) replication-independent ICL repair. Sister chromatids are depicted as blue and red lines. *C. elegans* proteins required for different pathways and specific steps are indicated.

Multiple conserved pathways recognize and repair different types of DNA damage. Some damaged bases can be directly repaired by specialized enzymes in a process referred to as damage reversal (DR). Base excision repair (BER) detects and excises a large variety of damaged bases, while leaving the phospho-ribose backbone intact (Beard *et al.* 2019). The resulting abasic site is converted into a single strand break (SSB) and, in turn, is repaired by the SSB repair (SSBR) pathway. Nucleotide excision repair (NER) typically acts on bases that carry bulky adducts such as those caused by the food toxins, aristolochic acid and aflatoxin, as well as on DNA intrastrand crosslinks such as thymidine dimers that form as a result of UV irradiation. NER acts by detecting a distortion of the double helix formed by these adducts, or interlinked bases, and by excising a short stretch of single-stranded DNA (ssDNA) carrying the damaged base(s) (Schärer 2013; Lans *et al.* 2019) (Figure  1A). DNA mismatch repair (MMR) is required to remove nucleotides misincorporated by replicative polymerases (Jiricny 2006; Pećina-Šlaus *et al.* 2020). When damaged bases fail to be repaired, translesion synthesis (TLS), a modality that involves specialized DNA polymerases capable of reading through damaged bases, provides a last resort for preventing DNA replication blockage, and the ensuing formation of double-strand breaks (DSBs) (Vaisman and Woodgate 2017). Given that TLS often leads to the incorporation of erroneous bases, “repair” by TLS can paradoxically be a source of mutagenesis, a phenomenon referred to as “error-prone repair.” DNA interstrand crosslinks (ICLs) are mended by DNA crosslink repair (CLR) modalities, including the Fanconi Anemia (FA) pathway (Stingele *et al.* 2017) (Figure  1B). 

**Figure 2:**
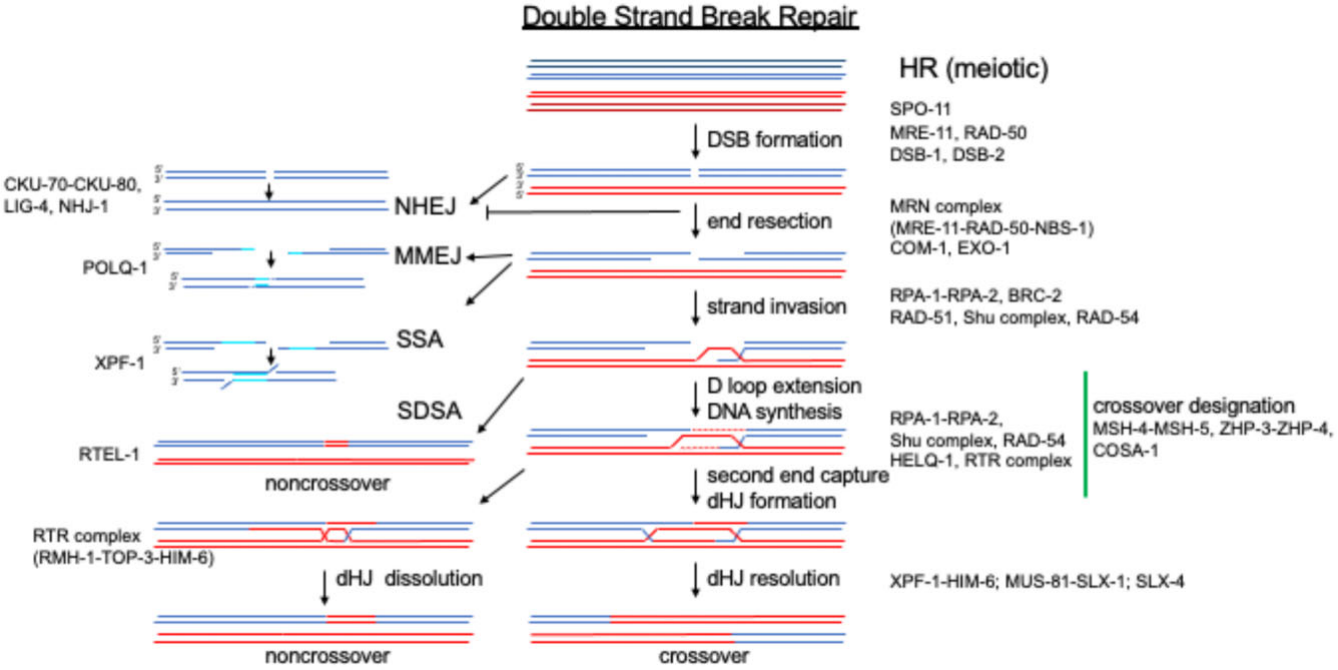
DSB repair. Homologous chromosomes are depicted as blue and red lines, the two sister chromatids are depicted in different shades on top; only a single sister for each homolog is depicted in the recombination process. *C. elegans* proteins required for different pathways and specific steps of DSB repair are indicated on the sides. DSBR, double-strand break repair; NHEJ, nonhomologous end joining; MMEJ, micromediated end joining; SSA, single strand annealing; SDSA, synthesis-dependent strand annealing; dHJ, double Holliday junction.

DSBs are one of the most deleterious DNA lesions and arise due to physical or chemical insult, or when the replication machinery encounters a DNA nick. DSBs are also intentionally induced during meiosis (Bergerat *et al.* 1997; Keeney *et al.* 1997; Dernburg *et al.* 1998), IgG class switching in mammals (Yu and Lieber 2019), and yeast mating type switching (Haber 2016). Depending on cell cycle stage and cell type, DSBs can be repaired by homologous recombination (HR), nonhomologous end joining (NHEJ), or other alternative pathways including microhomology mediated end joining (MMEJ) and single strand annealing (SSA) [for review, see Scully *et al.* (2019)] (Figure  2). NHEJ is the predominant repair pathway in most somatic cells and leads to the direct religation of broken DNA ends (Chang *et al.* 2017). MMEJ and SSA require short stretches of homology that are annealed, processed, and then ligated together, commonly leading to small deletions (Chang *et al.* 2017). In contrast to NHEJ, MMEJ, or SSA, HR relies on the use of an intact DNA molecule (the sister chromatid following S-phase of the cell cycle and either the nonsister or sister chromatid in prophase of meiosis I) to accurately repair the DSB without loss of genetic information. The current model for DSB repair by HR is largely based on the DSB repair (DSBR) model, originally formulated in 1983 (Szostak *et al.* 1983). DSBs are processed to reveal 3′ single-stranded tails, which are coated with RecA recombinases. Recombinases promote homology search and strand invasion of a homologous intact DNA duplex. Disassembly of recombinases from the heteroduplex DNA permits the initiation of DNA synthesis and second end capture; the resulting joint molecules (JMs) are processed by various enzymes to complete the repair reaction to generate crossover (CO) or non-CO (NCO) products. The principal mechanisms for HR are shared between mitosis and meiosis; in mitosis, HR is used to promote error-free repair, while meiotic HR is required for the exchange of genetic information between maternal and paternal chromosomes, essential for accurate chromosome segregation (Table  1).

**Table 1:** List of repair and recombination genes discussed in this chapter

Gene name	Ortholog	Molecular function (known or inferred^a^)	Role	References^b^
*agt-1*	MGMT	Alky guanyl transferase	Repair of O6 methyl-guanine	Kanugula and Pegg (2001)
*agt-2*	MGMT	Alky guanyl transferase	DNA damage signaling	Serpe *et al.* (2019)
*air-2*	AURKB	Protein kinase	Mitosis regulation, LEM-3 localization	Hong *et al.* (2018a)
*ama-1*	POLR2A	RNA polymerase I subunit	Transcription, failure to degrade upon UV leads to sensitivity	Astin *et al.* (2008)
*amx-1*	KDM1B	H3K4 demethylase	TC-NER	Wang *et al.* (2020b)
*apn-1*		Endonuclease	Incise AP sites/remove 3′-blocking lesions at DNA SSBs	Yang *et al.* (2012) and Papaluca *et al.* (2018)
*ash-2*	ASH2L	H3K4 methyltransferase^a^	TC-NER	Wang *et al.* (2020b)
*atl-1*	ATR	PI3-related protein kinase^a^	DNA damage checkpoint	Aoki *et al.* (2000) and Garcia-Muse and Boulton (2005)
*atm-1*	ATM	PI3-related protein kinase^a^	DNA damage checkpoint	Jones *et al.* (2012)
*brc-1*	BRCA1	E3 ubiquitin ligase; heterodimer with BARD1	IS repair/repair choice	Boulton *et al.* (2004) and Adamo *et al.* (2008)
*brc-2*	BRCA2	ssDNA-binding protein	RAD-51 loading/SSA	Petalcorin *et al.* (2006) and Ko *et al.* (2008)
*brd-1*	BARD1	E3 ubiquitin ligase; heterodimer with BRCA1	IS repair/repair choice	Boulton *et al.* (2004) and Adamo *et al.* (2008)
*cbp-1*	CREBBP	Histone acetyltransferase	Target of PRMT-5, inhibitor of *cep-1*	Yang *et al.* (2009)
*ccm-2*		PH-domain protein^a^	KRI-1 Adaptor	Chapman *et al.* (2019)
span[123] span[124]	VCP	Ubiquitin segregase	DNA damage-induced apoptosis	Ackermann *et al.* (2016)
*ced-1*	MEGF10	Scavenger receptor	Apoptosis	Zhou *et al.* (2001)
*ced-3*	CASP2	Cysteine-type endopeptidase, Caspase	Apoptosis	Yuan *et al.* (1993), Conradt and Xue (2005), Conradt (2009), and Bailly and Gartner (2013)
*ced-4*	APAF1	CARD domain protein	Apoptosis	Yuan and Horvitz (1992), Conradt and Xue (2005), Conradt (2009), and Bailly and Gartner (2013)
*ced-9*	BCL2	Bcl-2 like protein	Apoptosis	Hengartner *et al.* (1992), Conradt and Xue (2005), Conradt (2009), and Bailly and Gartner (2013)
*ced-13*	PUMA, NOXA	BH3 domain-only protein	Apoptosis	Schumacher *et al.* (2005a)
*cep-1*	TP53	Transcription factor	DNA damage checkpoint	Derry *et al.* (2001) and Schumacher *et al.* (2001)
*chd-3*	CHD-3/4	DNA helicase^a^	Repair choice	Turcotte *et al.* (2018)
*chk-1*	CHEK1	Protein kinase^a^	DNA damage checkpoint	Brauchle *et al.* (2003) and Jaramillo-Lambert *et al.* (2010)
*chk-2*	CHEK2	Protein kinase	Master regulator of meiotic prophase	MacQueen and Villeneuve (2001) and Kim *et al.* (2015)
*cku-70*	KU70	dsDNA-binding protein^a^	NHEJ	Clejan *et al.* (2006)
*cku-80*	KU80	dsDNA-binding protein^a^	NHEJ	Clejan *et al.* (2006)
*clk-2*	TELO2	Chaperonin^a^	DNA damage checkpoint	Ahmed *et al.* (2001)
*com-1*	CtIP	DNA binding/endonuclease^a^	DNA end resection	Penkner *et al.* (2007) and Lemmens *et al.* (2013)
*cosa-1*	CNTD1	Cyclin-related protein^a^	CO designation	Yokoo *et al.* (2012)
*cra-1*	NAA25	Noncatalytic subunit of NatB acetyltransferase^a^	DSB formation	Smolikov *et al.* (2008) and Gao *et al.* (2015)
*csa-1*	ERCC8	WD-40 repeat protein; ubiquitin-protein transferase^a^	TC-NER	Babu *et al.* (2014)
*csb-1*	ERCC6	DNA binding; ATPase^a^	TC-NER/CLR	Babu *et al.* (2014)
*daf-16*	FOXO1/FOXO3/FOXO4	Transcription factor	TC-NER	Bianco and Schumacher (2018)
*ddb-1*	DDB1	Adaptor for CUL4-based E3 ubiquitin ligase	NER	Arczewska *et al.* (2013)
*dna-2*	DNA2	Helicase/nuclease	Resection	Ryu and Koo (2017)
*dog-1*	FANCJ	DNA helicase^a^	CLR	Wu *et al.* (2008) and Kruisselbrink *et al.* (2008)
*dot-1.1*	DOTL1	Histone H3K79 methyltransferase^a^	Meiotic checkpoint	Lascarez-Lagunas *et al.* (2020)
*dsb-1*	REC114	Spo11 accessory protein^a^	DSB formation	Stamper *et al.* (2013)
*dsb-2*	REC114	Spo11 accessory protein^a^	DSB formation	Rosu *et al.* (2013)
*dsb-3*	MEI4	Spo11 accessory protein^a^	DSB formation	Hinman *et al.* (2021)
*dss-1*	SEM1	BRCA2 regulatory protein	RAD51 loading/SSA	Martin *et al.* (2005)
*dut-1*	DUT	dUTP diphosphatase activity^a^	Removal of dUTP	Dengg *et al.* (2006)
*dvc-1*	SPRTN	DNA metalloprotease^a^	Removal of DNA-protein crosslinks	Stingele *et al.* (2016)
*eel-1*	HUWE1	HECT-domain E3 ligase^a^	DNA damage-induced apoptosis	Ross *et al.* (2011)
*egl-1*	PUMA, NOXA	BH3 domain-only protein	Apoptosis	Conradt and Horvitz (1998)
*egl-15*	FGFR1/FGFR3	Protein kinase	Induction of IFE-4 for DNA damage cell cycle arrest	Ou *et al.* (2019)
*egl-17*	FGF17/FGF18/FGF8	Fibroblast growth factor^a^	Induction of IFE-4 for DNA damage cell cycle arrest	Ou *et al.* (2019)
*egl-27*	RERE	Transcription factor	TC-NER	Mueller *et al.* (2014)
*eme-1*	EME1	Structure-specific endonuclease; heterodimer with MUS-81^a^	HJ resolution	Saito *et al.* (2013)
*ercc-1*	ERCC1	Regulatory subunit of XPF		Agostinho *et al.* (2013)
*exo-1*	EXO1	Exonuclease^a^	Resection	Lemmens *et al.* (2013), Yin and Smolikove 2013), and Girard *et al.* (2018)
*exo-3*	APEX1	Exonuclease	Incise AP sites/remove 3′ blocking lesions at DNA SSBs	Yang *et al.* (2012) and Papaluca *et al.* (2018)
*fan-1*	FAN1	Structure-specific nuclease^a^	CLR	Kratz *et al.* (2010)
*fcd-2*	FANCD2	DNA binding^a^	CLR	Dequen *et al.* (2005a), Collis *et al.* (2006), and Lee *et al.* (2007)
*fnci-1*	FANCI	DNA binding^a^	CLR	Lee *et al.* (2010b)
*fncm-1*	FANCM	DNA helicase^a^	CLR	Lee *et al.* (2010b)
*fsn-1*	FBXO45	F-box of cullin3^a^	Modulates apoptotic response through controlling CEP-1 protein levels	Gao *et al.* (2008)
*gcna-1*	GCNA	Protease^a^	Removal of DNA–topoisomerase crosslinks	Borgermann *et al.* (2019), Bhargava *et al.* (2020), and Dokshin *et al.* (2020)
*gei-17*	PIAS1/PIAS2/PIAS3	SUMO ligase	Regulation of TLS	Kim and Michael (2008)
*gen-1*	GEN1	HJ resolvase	Resolution of JMs/checkpoint signaling	Bailly *et al.* (2010)
*gld-1*	QKI	RNA-binding protein	Translational repression of *cep-1*	Schumacher *et al.* (2005b)
*glp-1*	NOTCH1/2/3	RNA polymerase II binding	Required for mitotic germ cell fate; inhibits ATM checkpoint kinase	Vermezovic *et al.* (2015)
*hcp-3*	CENPA	Centromeric histone^a^	Enrichment at nuclear periphery in response to DNA damage	Lawrence *et al.* (2015)
*hcp-6*	NCAPD3	Condensin II complex subunit	Chromosome condensation	Stear and Roth (2002) and Yeong *et al.* (2003)
*hrde-1*		RNA binding^a^	Required for secondary 22 nt RNAi	McMurchy *et al.* (2017)
*helq-1*	HELQ	Helicase	RAD-51 disassembly	Ward *et al.* (2010)
*hif-1*	EPAS1/HIF1A	Transcription factor	Hypoxia blockage of DNA damage-induced apoptosis	Sendoel *et al.* (2010)
*him-5*		Chromatin associated	DSB formation	Meneely *et al.* (2012) and Chung *et al.* (2015)
*him-6*	BLM	RecQ-like helicase	HJ dissolution/HJ resolution	Zetka and Rose (1995)
*him-14/msh-4*	MSH4	DNA-dependent ATPase; heterodimer with MSH5^a^	CO formation	Kelly *et al.* (2000)
*him-17*	THAP	Chromatin associated	DSB formation	Reddy and Villeneuve (2004)
span[17527] span[17528]	SLX4	Structure-specific endonuclease scaffold	HJ resolution	Agostinho *et al.* (2013), O’Neil *et al.* (2013), and Saito *et al.* (2013)
*hpl-1*	CBX3	Heterochromatin-binding protein	Heterochromatin function	Johnson *et al.* (2013) and McMurchy *et al.* (2017)
*hpl-2*	CBX3	Heterochromatin-binding protein	Heterochromatin function	Johnson *et al.* (2013) and McMurchy *et al.* (2017)
*hpr-17*	RAD17	Chromatin associated; clamp loader^a^	DNA damage checkpoint	Boerckel *et al.* (2007)
*hsp-70*	HSPA6 (HSP70)	Chaperon, heat shock protein^a^	DNA damage-induced apoptosis	Bailly *et al.* (2019)
*hsr-9*	TP53BP1	Histone binding^a^	DNA damage checkpoint	Ryu *et al.* (2013)
*hus-1*	HUS1	Chromatin associated; clamp loader^a^	DNA damage checkpoint	Hofmann *et al.* (2002)
*icap-1*	ITGB1BP1	PH-domain protein^a^	KRI-1 adaptor; DNA damage-induced apoptosis	Chapman *et al.* (2019)
*ife-4*	EIF4E2	Translation initiation factor	Germ cell precursor cell cycle arrest upon DNA damage	Ou *et al.* (2019)
*jmjd-1.1*	KDM7A	Histone lysine demethylase^a^	CLR	Lee *et al.* (2015)
*klf-3*	KLF1	Transcription factor	Regulates Zn2+ transport for regulation of apoptosis	Chapman *et al.* (2019)
*kri-1*	KRIT1/CCM1		DNA damage-induced apoptosis	Chapman *et al.* (2019)
*ksr-1*	KSR1	Scaffold for MAP kinase signaling	Potential target of Zn2+ inhibition of MAP kinase signaling	Chapman *et al.* (2019)
*lem-3*	ANKLE1	Ankyrin repeat and LEM domain containing nuclease	HR processing/resolution of chromatin bridges	Hong *et al.* (2018a)
*let-418*	CHD3/4	DNA helicase^a^	Repair choice	Turcotte *et al.* (2018)
*lig-4*	LIG4	DNA ligase IV^a^	NHEJ	Clejan *et al.* (2006)
*lin-35*	RBL1/RBL2	Transcriptional corepressor	Germ cell apoptosis	Schertel and Conradt (2007)
*lin-61*	SFMBT2	Chromatin associated	Microsatellite stability	Johnson *et al.* (2013) and McMurchy *et al.* (2017)
*lip-1*	DUSP6/7	MAP kinase phosphatase^a^	Negative regulator of MAP kinase signaling	Rutkowski *et al.* (2011)
*mad-2/mdf-2*	MAD2L1	Spindle checkpoint	SAC; DNA repair	Lawrence *et al.* (2015)
*mek-5*	KHDRBS2	Protein kinase and RNA binding^a^	MAP kinase signaling	Chapman *et al.* (2019)
*mekk-3*	MAP kinase kinase	Protein kinase^a^	MAP kinase signaling	Chapman *et al.* (2019)
*met-2*	SETDB1/SETDB2	H3K9 mono/dimethyltransferase^a^	Heterochromatin formation	Bessler *et al.* (2010)
*mlh-1*	MLH1	ATPase^a^	DNA MMR	Degtyareva *et al.* (2002) and Tijsterman *et al.* (2002)
*mpk-1*	MAPK1	MAP kinase	MAP kinase signaling	Eberhard *et al.* (2013)
*mpk-2*	MAPK7	MAP kinase	MAP kinase signaling	Chapman *et al.* (2019)
*mre-11*	MRE11	3′–5′ DNA exonuclease/ssDNA endonuclease^a^; MRN complex	DSB formation/resection	Chin and Villeneuve (2001), Rinaldo *et al.* (2002), and Hayashi *et al.* (2007)
*mrt-1*	DCLRE1B	Exonuclease	Telomere maintenance/NER/CLR	Meier *et al.* (2009)
*mrt-2*	RAD1	Subunit of 9–1–1 clamp loader complex	DNA damage checkpoint, telomere replication	Gartner *et al.* (2000)
*msh-2*	MSH2	DNA binding^a^/MutSa complex	DNA MMR	Degtyareva *et al.* (2002)
*msh-5*	MSH5	DNA-dependent ATPase^a^; MutSg heterodimer with MSH4	CO formation	Kelly *et al.* (2000)
*msh-6/msh-3*	MSH6	DNA-binding^a^MutSa complex	DNA MMR	Tijsterman *et al.* (2002)
*mus-81*	MUS81	Structure-specific endonuclease; heterodimer with EME1	HJ resolution	Agostinho *et al.* (2013) and Saito *et al.* (2013)
*mys-1*	KAT5	H4 acetyltransferase^a^	DNA damage checkpoint	Couteau and Zetka (2011)
*nbs-1*	NBS1	Forkhead-associated domain protein^a^; MRN complex	Resection	Girard *et al.* (2018)
*ncc-1/cdk-1*	CDK1	Cyclin-dependent protein kinase	PhosphoTyr15 marker of G2	Moser *et al.* (2009) and Craig *et al.* (2012)
*ndx-1*	NUDT18	Hydrolase^a^	Cleavage of 8-oxo-dGDP	Sanada *et al.* (2011)
*ndx-2*	NUDT5	Hydrolase^a^	Cleavage of 8-oxo-dGDP	Sanada and Zhang-Akiyama (2014)
*ndx-4*	NUDT2	Hydrolase	Cleavage of 8-oxo-dGTP	Arczewska *et al.* (2011)
*nhj-1*			NHEJ	Vujin *et al.* (2020)
*nth-1*	NTHL1	Glycosylase	Removes 5-hmU	Papaluca *et al.* (2018)
*parg-1/pme-3*	BPHL	Poly(ADP-ribose) glycohydrolase	DNA repair	St-Laurent *et al.* (2007) and Bae *et al.* (2020)
*parg-2/pme-4*	BPHL	Poly(ADP-ribose) glycohydrolase	DNA repair/meiotic recombination	St-Laurent *et al.* (2007) and Bae *et al.* (2020)
*parp-1/pms-1*	PARP1	Poly(ADP-ribose) polymerase	DNA repair	Gagnon *et al.* (2002) and Dequen *et al.* (2005b)
*parp-2/pms-2*	PARP2	Poly(ADP-ribose) polymerase	DNA repair	Gagnon *et al.* (2002) and Dequen *et al.* (2005b)
*pch-2*	TRIP13	AAA-ATPase	Synapsis checkpoint	Bhalla and Dernburg (2005) and Deshong *et al.* (2014)
*pgl-1*		Endoribonuclease	P granule organization; required for DNA damage-induced apoptosis	Raiders *et al.* (2018) and Min *et al.* (2019)
*pmk-1*	MPK11/MPK14	MAP kinase	Ceramide-dependent apoptosis	Yang *et al.* (2021)
*pms-2*	PMS2	ATPase^a^	DNA MMR	Degtyareva *et al.* (2002) and Tijsterman *et al.* (2002)
*polh-1*	POLH (eta)	DNA polymerase^a^	TLS	Roerink *et al.* (2012)
*polk-1*	POLK (kappa)	DNA polymerase^a^	TLS	Roerink *et al.* (2012)
*polq-1*	POLQ (theta)	DNA polymerase^a^	MMEJ	Koole *et al.* (2014), Roerink *et al.* (2014), and van Schendel *et al.* (2016)
*prg-1*	PIWIL1	21U-RNA-binding activity	Heterochromatin function	McMurchy *et al.* (2017)
*prmt-5*	PRMT5	Protein arginine methyltransferase	Negative regulator of *cep-1*	Yang *et al.* (2009)
*rad-50*	RAD50	SMC ATPase^a^; MRN complex	DSB formation/resection	Chin and Villeneuve (2001), Rinaldo *et al.* (2002), and Hayashi *et al.* (2007)
*rad-51*	RAD51	RecA recombinase	Homology search/strand invasion	Rinaldo *et al.* (2002)
*rad-54*	RAD54	DNA translocase	D-loop remodeling/RAD51 disassembly	Mets and Meyer (2009) and Ward *et al.* (2010)
*raf-1/lin-45*	ARAF	RAS GTPase binding	Potential target of Zn2+ inhibition of MAP kinase signaling	Yoder *et al.* (2004) and Jirakulaporn and Muslin (2004)
*rcq-5*	RECQL5	Helicase^a^	RAD-51 disassembly	Jeong *et al.* (2003)
*rec-1*			Crossover distribution	Rose and Baillie (1979)
*rev-1*	REV1	DNA polymerase^a^	TLS/CLR	Oh *et al.* (2020)
*rev-3*	REV3L (zeta)	DNA polymerase^a^	TLS	Roerink *et al.* (2012), van Bostelen and Tijsterman (2017), and van Bostelen *et al.* (2020)
*rfs-1*	RAD51D	Shu mediator complex	Strand invasion/D-loop formation/RAD51 disassembly	Ward *et al.* (2007), Yanowitz (2008), Taylor *et al.* (2015), and McClendon *et al.* (2016)
*rip-1*	RAD51 paralog	Shu mediator complex	Strand invasion/D-loop formation/RAD51 disassembly	Ward *et al.* (2007), Yanowitz (2008), Taylor *et al.* (2015), and McClendon *et al.* (2016)
*rmh-1*	RMI1	DNA-binding activity^a^; RTR complex	HJ dissolution/CO formation	Wicky *et al.* (2004), Schvarzstein *et al.* (2014), and Jagut *et al.* (2016)
*rmif-2*	RMI2	RTR complex	HJ dissolution/CO formation	Velkova *et al.* (2021)
*rnf-113*	RNF113A/RNF113B	Ubiquitin transferase	CLR	Lee *et al.* (2013)
*rpa-1*	RPA1	ssDNA-binding protein	Pre- and post-RAD-51 role in HR	Koury *et al.* (2018) and Hefel *et al.* (2020)
*rpa-2*	RPA2	ssDNA-binding protein	Pre- and post-RAD-51 role in HR	Koury *et al.* (2018) and Hefel *et al.* (2020)
*rpa-4*	RPA2	ssDNA-binding protein	Pre- and post-RAD-51 role in HR	Koury *et al.* (2018) and Hefel *et al.* (2020)
*rpoa-2*	POLR1B	RNA polymerase I subunit^a^	Apoptosis	Eberhard *et al.* (2013)
*rtel-1*	RTEL1	Helicase	SDSA, HR	Barber *et al.* (2008)
*ruvb-1/2*	RUVBL1	Helicase^a^	Chromatin decompaction	Wong *et al.* (2018)
*scc-2*	SCC2	Cohesin loading^a^	Checkpoint-induced apoptosis	Lightfoot *et al.* (2011)
*scc-3*	STAG1/STAG3	Cohesin complex	Checkpoint-induced apoptosis	Lightfoot *et al.* (2011)
*set-2*	SETD1A/SETD1B	H3K4 methyltransferase	TC-NER	Herbette *et al.* (2017)
*set-16*	KMT2C	H3K4 methyltransferase	TC-NER	Wang *et al.* (2020b)
*set-25*	SUV39h1/SUV39h2/G9a	H3K9 trimethyltransferase	Heterochromatin formation	Zeller *et al.* (2016) and McMurchy *et al.* (2017)
*sir-2.1*	SIRT1	Histone deacetylase	DNA damage-induced apoptosis	Greiss *et al.* (2008b)
*slx-1*	SLX1A	Endonuclease	HJ resolution	Agostinho *et al.* (2013) and Saito *et al.* (2013)
*smc-5*	SMC5	SMC; ATP-binding activity^a^; heterodimer with SMC6	IS repair	Boulton *et al.* (2004), Bickel *et al.* (2010), and Wolters *et al.* (2014)
*smc-6*	SMC6	SMC; ATP-binding activity^a^; heterodimer with SMC5	IS repair	Boulton *et al.* (2004), Bickel *et al.* (2010), and Wolters *et al.* (2014)
*smg-1*	SMG1	PI3-related kinase^a^	NMD and DNA repair	González-Huici *et al.* (2017)
*smrc-1*	SMARCAL1	SWI/SNF ATPase^a^	DNA repair	Yang *et al.* (2019)
*spo-11*	SPO11	Topoisomerase^a^	DSB formation	Dernburg *et al.* (1998)
*spr-5*	KDM1A	H3K4 demethylase	TC-NER	Wang *et al.* (2020b)
*sun-1*	SPAG4/SUN3/SUN5	SUN domain protein	Chromosome pairing	Malone *et al.* (2003) and Penkner *et al.* (2009)
*sws1*	SWIM	Shu mediator complex	Strand invasion/D-loop formation/RAD51 disassembly	Ward *et al.* (2007), Yanowitz (2008), Taylor *et al.* (2015), and McClendon *et al.* (2016)
*syp-1*		SC central region component	Phospho-dependent regulation in response to IR	Garcia-Muse *et al.* (2019)
*top-2*	TOPO2	Topoisomerase^a^	Chromatin decompaction	Jaramillo-Lambert *et al.* (2016) and Bhandari *et al.* (2020)
*top-3*	TOPO3	Topoisomerase^a^; RTR complex	HJ dissolution	Wicky *et al.* (2004)
*tyr-2*	DCT/TYR	Tyrosinase	Hypoxia blockage of DNA damage-induced apoptosis	Sendoel *et al.* (2010)
*udf-2*	UBE4B	E4 Ubiquitin ligase	DNA damage-induced apoptosis	Ackermann *et al.* (2016)
*ulp-3*	SENP8	Cysteine-type peptidase (NEDD8)^a^	DNA damage-induced apoptosis	Bailly *et al.* (2019)
*unc-58*		Potassium channel	Muscle contraction; reversion assay	Harris *et al.* (2006)
*unc-83*		KASH domain protein	Nuclear migration	McGee *et al.* (2006)
*unc-84*	SUN2	SUN domain protein	Nuclear migration/CLR	Malone *et al.* (2003), Penkner *et al.* (2009) and Lawrence *et al.* (2016)
*unc-93*	UNC93A	Potassium channel regulator	Muscle contraction; reversion assay	(Degtyareva *et al.* 2002; Tijsterman *et al.* 2002)
*ung-1*	UNG	DNA glycosylase	Removes uracil from DNA	Nakamura *et al.* (2008) and Skjeldam *et al.* (2010)
span[132] span[133]	WDR5	WD repeat protein; RNA polymerase II binding	TC-NER	Wang *et al.* (2020b)
*wrn-1*	WERNER	Helicase	Resection	Ryu and Koo (2017)
*wwp-1*	ITCH/WWP1/WWP2	Ubiquitin conjugation	TC-NER	Astin *et al.* (2008)
*xnd-1*		AT-hook containing protein	DSB formation	Reddy and Villeneuve (2004), Wagner *et al.* (2010), Meneely *et al.* (2012), Gao *et al.* (2015), and Chung *et al.* (2015)
*xpa-1*	XPA	DNA-binding activity^a^	NER/CLR	Wilson *et al.* (2017)
*xpc-1*	XPC	ssDNA-binding activity^a^	NER/CLR	Wilson *et al.* (2017)
*xpf-1*	ERCC4	Endonuclease	CLR/HJ resolution/SSA	Wilson *et al.* (2017)
*xpg-1*	ERCC5	Endonuclease^a^	NER/CLR	Wilson *et al.* (2017)
*zhp-1*	HEI10	E3 ligase^a^ heterodimer with ZHP-2	Negatively regulates CO designation/required for CO maturation	Zhang *et al.* (2018)
*zhp-2*	HEI10	E3 ligase^a^; heterodimer with ZHP-1	Negatively regulates CO designation/required for CO maturation	Zhang *et al.* (2018)
*zhp-3*	RNF212	E3 ligase^a^; heterodimer with ZHP-4	CO designation	Bhalla *et al.* (2008)
*zhp-4*	RNF212	E3 ligase^a^; heterodimer with ZHP-3	CO designation	Zhang *et al.* (2018) and Nguyen *et al.* (2018)
*zpf-1*	MLLT10	Chromatin and methylated histone binding	Checkpoint signaling	Lascarez-Lagunas *et al.* (2020)
*ztf-8*	RHINO	DNA-binding protein^a^	Checkpoint signaling	Kim and Colaiácovo (2014)
*zyg-12*	HOOK1	KASH domain protein	Chromosome pairing/CLR	Lawrence *et al.* (2016)

a Inferred function.

b References are not comprehensive.

DNA damage response (DDR) checkpoints are required to transiently halt cell cycle progression to allow for DNA repair or to eliminate damaged cells by triggering apoptosis (Jackson and Bartek 2009). The activation of DNA damage checkpoints requires the conserved phosphatidylinositol 3 (PI3)-kinase like family of protein kinases, ATM and ATR, which act at the apex of signaling cascades, sensing resected DSBs, which also serve as a substrate for HR, and ssDNA, which accumulates when replication is compromised (Jackson and Bartek 2009; Blackford and Jackson 2017). An important effector of checkpoint signaling is the conserved p53 transcription factor, which promotes cell cycle arrest or apoptosis depending on cell type and the extent of DNA damage (Vousden and Lane 2007; Lane and Levine 2010).

*C.* *elegans* mutants hypersensitive to ionizing radiation (IR) and UV treatment were first described in 1982 (Hartman and Herman 1982). Almost 40 years later an extensive literature on *C. elegans* DNA repair and DNA damage exists. We begin by reviewing the attributes of *C. elegans* that facilitate studies of DNA repair, recombination, and checkpoint signaling (see *Studying repair, recombination, and checkpoint signaling in C. elegans*) and then discuss DNA damage repair (see *DNA repair*). In *DSB repair*, we turn our attention to recombination, with a focus on HR and the associated function of *C. elegans* proteins required for DSB formation and processing in the germ line. We concentrate on the DNA events of recombination and refer the reader to the Meiosis Chapter for insight into the associated chromosomal events unique to meiotic recombination (Hillers *et al.* 2017). We will then discuss the signaling pathways that monitor DNA damage or aberrant recombination (see *DDR, checkpoint signaling, fail-safe mechanisms, and apoptosis induction*) and in the final section, focus on recent studies that have used *C. elegans* to define mutational signatures, critical for understanding mutations associated with disease and aging (see *Using C. elegans to define mutational signatures*).

## Studying repair, recombination, and checkpoint signaling in *C. elegans*

**Figure 3:**
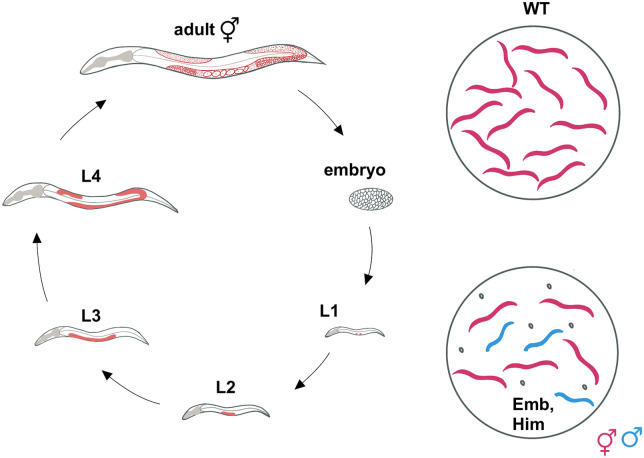
*C. elegans* life cycle and plate phenotypes of recombination mutants. Left: cartoon of *C. elegans* embryo, larval stages (L1–L4), and adult hermaphrodite. Germline cells are shown in red, gray marks the pharynx, required for both respiratory and digestive functions. Right: plate phenotypes of wild-type and recombination defective mutants, which are Emb and Him. Hermaphrodites are shown in magenta, males in blue, and unhatched embryos in black.

*C.* *elegans* is a small (1 mm), free-living self-fertilizing nematode that is easy to maintain, propagate, and store in the laboratory. Worms are grown on simple medium seeded with *E. coli*, which serves as a food source. At 20°C, under optimal conditions, *C. elegans* has a 3½ day life cycle (from embryo to adult), which comprises the embryonic stage, four larval stages (L1–L4), and adulthood (Figure  3).

### DNA repair and checkpoint assays

A general advantage of *C. elegans* is that, in contrast to mammalian systems, mutations of the vast majority of DNA repair and checkpoint factors do not have overt developmental phenotypes, greatly facilitating genetic analysis. The exception are repair genes that have a critical role in meiotic recombination; however, even for those repair mutants, homozygous filial (F1) lines derived from heterozygous parents can be studied as they develop normally into adults but produce dead progeny (embryonic lethality—Emb phenotype; Figure  3) in the following generation. A variety of assays are used to assess the status of DNA repair and checkpoint signaling in *C. elegans* and several methods chapters are available (Gartner *et al.* 2004; Craig *et al.* 2012; García-Muse 2021). In a nutshell, survival assays gauge the efficiency of DNA repair mechanisms in germ cells by scoring the number (brood size) and survival of embryos laid. Typically, late L4 or early adult animals are treated with genotoxic agents such as UV, IR, or mutagenic drugs, and the survival rate of embryos laid 24–36 h later is assayed. In this time frame, meiotic cells in the pachytene stage differentiate into gametes, fuse, and form embryos. If sensitivity of mitotically proliferating germ cells is to be scored, the survival of embryos laid ∼48–72 h after exposure to genotoxic agents is assayed; 48–72 h is the time it takes for germ cells in the proliferative zone to transit through the germ line (Crittenden *et al.* 2006; Jaramillo-Lambert *et al.* 2007). The aforementioned assay can also be adapted to directly score for chromosomal fragmentation in meiotic diplotene cells (Craig *et al.* 2012) (see below). In addition, a reduction in the rate of germ cell proliferation, measured as the average number of embryos laid over a set period, provides an indication of repair defects. Other commonly used assays score for the effects of DNA damage by measuring developmental delays and abnormal development. For instance, NHEJ activity is gauged by treating late stage embryos with IR, and monitoring the pace of development, as well as developmental abnormalities, such as movement defects and misshapen vulval structures. L1 stage animals, which are easily obtained in large quantities by filtering or by allowing embryos to hatch without food, are also commonly treated with genotoxic agents (Bailly *et al.* 2010; Craig *et al.* 2012). This assay targets the proliferating germ line, which massively expands during larval development; if proliferation is impaired by genotoxic agents, the resulting worms are sterile. At the same time developmental delay or arrest can be determined. Typically, hypersensitivity to IR leads to sterile germ lines without affecting the rate of development. Treatment with UV or alkylating agents can also lead to developmental delay or arrest, especially in DNA repair defective mutants (Lans *et al.* 2010; Craig *et al.* 2012; Mueller *et al.* 2014; Wilson *et al.* 2017). These later phenotypes are highly pronounced in transcription-coupled NER mutants, in line with DNA damage-induced transcriptional deficiency causing those phenotypes.

To determine the relative contribution of various DSB repair modalities in somatic cells, a reporter containing an 18-nucleotide SceI restriction endonuclease site and two nonfunctional copies of LacZ was developed. HR and SSA, but not NHEJ, can restore a functional LacZ following DNA breakage by SceI cleavage (Pontier and Tijsterman 2009). Finally, the mutator phenotype associated with DNA repair defective strains can be measured by determining reversion rates of immobile *unc-58(e665)* (Harris *et al.* 2006) or uncoordinated and egg-laying defective *unc-93(e1500)* mutants (Degtyareva *et al.* 2002; Tijsterman *et al.* 2002). More recently, mutational profiles were directly determined by whole genome sequencing (see *Using C. elegans to define mutational signatures*).

**Figure 4:**
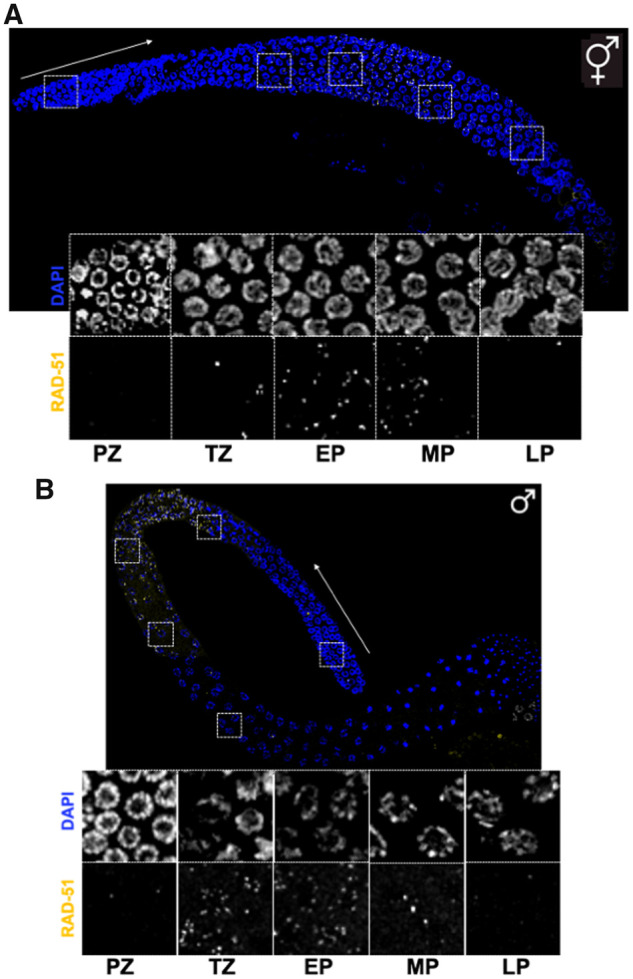
RAD-51 foci in the hermaphrodite and male germ line. (A) Half projections of hermaphrodite (top) and (B) male (bottom) gonads, fixed, dissected and stained with anti-RAD-51 antibody (yellow) and counterstained with DAPI (blue). Insets show higher magnification of indicated regions for both RAD-51 and DAPI. PZ, proliferative zone; TZ, transition zone; EP, early pachytene; MP, midpachytene; LP, late pachytene. Scale bar = 20μm.

Repair activity is also analyzed by determining the number and kinetics of key DNA repair foci cytologically, the most important factor being the RAD-51 recombinase, whose presence in foci is indicative of ongoing HR (Alpi *et al.* 2003; Colaiácovo *et al.* 2003; García-Muse 2021) (Figure  4). Additionally, the HR proteins, BRC-1/BRCA1, BRD-1/BARD1, and BRC-2/BRCA2, also form foci (Martin *et al.* 2005; Polanowska *et al.* 2006; Janisiw *et al.* 2018; Li *et al.* 2018). Other commonly monitored factors include a component of replication protein complex A, replication protein A (RPA-1), which marks ssDNA (Polanowska *et al.* 2006; Moser *et al.* 2009; Lee *et al.* 2010a), HUS-1, a readout of checkpoint activation (Hofmann *et al.* 2002), and the FCD-2/FANCD2 protein, which is recruited to ICLs (Collis *et al.* 2006; Lee *et al.* 2007). Foci are typically scored using dissected gonads and specific antibodies or reporter gene fusions. Procedures to analyze repair foci in L1 germ lines were recently summarized (Ou and Schumacher 2021). DNA strand breaks in mitotically dividing germ cells or dissociated whole worms have also been measured using COMET assays, where broken DNA forms a characteristic comet shape following gel electrophoresis (Imanikia *et al.* 2016; Park *et al.* 2016). Microbeam irradiation provides an exciting possibility to induce localized damage, restricted to suborganismal or subcellular compartments (Tang *et al.* 2016; Torfeh *et al.* 2019). Irradiation of a small number of nuclei by a laser beam can be used to induce DSBs to precisely determine repair kinetics by examining the timing of appearance of cytological markers (Koury *et al.* 2018).

To assess DNA damage checkpoint activation, the extent of DNA damage-induced germ cell apoptosis is measured by directly scoring apoptotic corpses under Nomarski optics, or by staining apoptotic cells with acridine orange or SYTO 12, dyes that stain nucleic acids in a low pH environment associated with phagocytized apoptotic corpses (Craig *et al.* 2012; Lant and Derry 2014). In addition, phagocytic markers, including CED-1:YFP, CED-1::GFP, and ACT-5::YFP, have been used to measure apoptosis (Schertel and Conradt 2007; Lant and Derry 2014). As DNA damage checkpoint activation involves the p53-dependent transcriptional induction of the proapoptotic BH3-only genes *ced-13* and *egl-1*, mRNA expression can be measured directly by RT-PCR assays or by using reporters (Hofmann *et al.* 2002; Craig *et al.* 2012; Doll *et al.* 2019). Checkpoint-induced cell cycle arrest is monitored by scoring for the number of mitotic germ cells in a defined area; cell cycle arrested cells continue to grow, resulting in a smaller number of larger cells (Gartner *et al.* 2000, 2004). DNA damage-induced checkpoint activation can also be measured by labeling with an antibody that recognizes a conserved phospho-epitope of the checkpoint kinase CHK-1 (Jaramillo-Lambert *et al.* 2010), while labeling with an antibody that recognizes a conserved phospho-epitope on the worm NCC-1/CDK-1 protein (homologous to mammalian CDK1 tyrosine-15) can highlight cells undergoing G2 cell cycle arrest in the mitotic zone of the germ line (Moser *et al.* 2009; Craig *et al.* 2012).

### Germline biology and analysis of meiotic recombination progression

In the laboratory, *C. elegans* exist predominantly as hermaphrodites, initially producing sperm at the L3/L4 molt, switching exclusively to oogenesis at the L4/adult molt. Males arise spontaneously at a rate of ∼0.2% due to meiotic X chromosome nondisjunction and only produce sperm (Hodgkin *et al.* 1979). Sex is determined by the X to autosome ratio; hermaphrodites are XX while males are XO (see Sex Determination Chapter). Errors in meiosis lead to the production of nullo X gametes that, when fused to normal X containing gametes, generate male worms. A high incidence of males (Him) among self-progeny has been used as a simple phenotypic readout for elevated chromosome nondisjunction, and has led to the identification of several genes important for meiotic recombination (Hodgkin *et al.* 1979; Zalevsky *et al.* 1999; Kelly *et al.* 2000). Chromosome nondisjunction of autosomes leads to the Emb phenotype, another hallmark of meiotic recombination mutants. Many mutants that primarily affect recombinational repair and checkpoint signaling have weak Him and Emb phenotypes, indicating functional overlap and redundancies between meiotic recombination, recombinational repair, and DNA damage checkpoint signaling.

The germ line is the major tissue where DNA repair and damage response pathways are studied. At the same time, DSBs occur naturally during meiosis and are processed by recombination pathways. The germ line makes up approximately half of the cells in the adult worm, providing a rich source for analyses. The two U-shaped gonads in the hermaphrodite serve as production lines for gamete formation: germ cells representing every stage of meiotic prophase are arranged in a spatiotemporal pattern (Figure  4). The polarity of the gonad is defined by the distal tip cell (DTC), which migrates during larval development and defines the morphology of the mature gonad. Adjacent to the DTC, at the distal end of the gonad, proliferating germ cells divide mitotically. As cells move proximally away from the DTC, they enter meiosis and undergo a single round of DNA replication (meiotic S-phase). Chromosome pairing (*i.e.*, the close alignment of homologous chromosomes) occurs in the transition zone (leptotene/zygotene) and leads to a distinctive crescent shape of the chromatin, as visualized by DAPI (4′,6-diamidino-2-phenylindole) staining. Unlike many organisms, in *C. elegans*, pairing can occur independently of meiotic recombination (Dernburg *et al.* 1998) and relies on specialized sequences at chromosome ends, called pairing centers, that drive chromosome movement critical for initial alignment of homologous chromosomes (Villeneuve 1994; Penkner *et al.* 2009; Baudrimont *et al.* 2010; Wynne *et al.* 2012). Pairing is stabilized by the assembly of the meiosis-specific synaptonemal complex (SC) between homologous chromosomes at pachytene (MacQueen *et al.* 2002; Colaiácovo *et al.* 2003). DSB formation is initiated in the transition zone/early pachytene; DSB processing occurs throughout pachytene. Cells exiting pachytene disassemble the SC and resolve any remaining recombination intermediates. Chromosome remodeling around a single CO, the chiasma, begins at late pachytene and results in a cruciform structure at diakinesis (Nabeshima *et al.* 2004). Oocytes remain in diakinesis until they are fertilized by sperm stored in the spermatheca. Fertilization triggers the onset of the oocyte meiotic divisions, fusion of the oocyte and sperm nuclei, and initiation of the early embryonic divisions.

**Figure 5:**
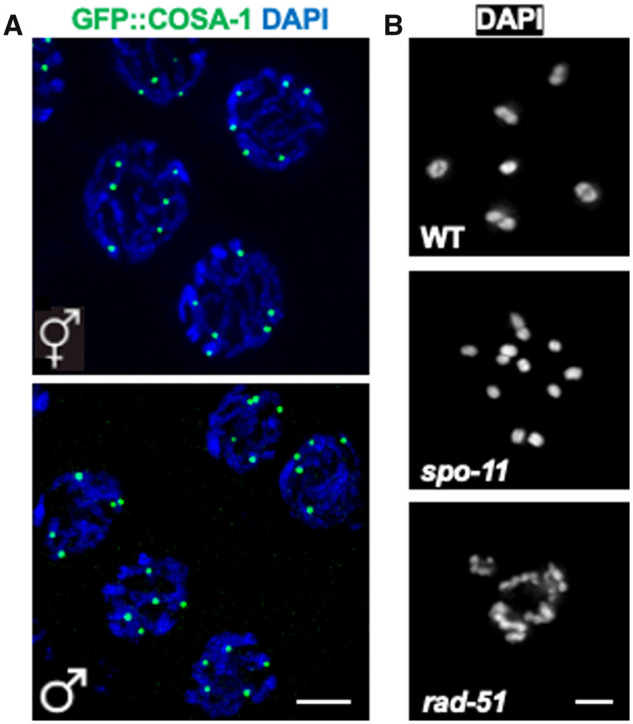
IH crossover assays. (A) Half projections of late pachytene nuclei in hermaphrodite (top) and male (bottom) showing GFP::COSA-1 (green), a crossover designation marker, and DAPI (blue). There are six COSA-1 foci in hermaphrodites, one for each homologous chromosome pair and five in males, for the five pairs of autosomes. (B) Diakinesis nucleus of WT, *spo-11(ok79)*, and *rad-51(ok2218)* mutants stained with DAPI to visualize the individual chromosomes—in wild type there are six bivalents, connected by chiasmata. In the *spo-11* mutant there are 12 univalents due to absence of DSBs and crossovers, while in *rad-51* there are chromosome aggregates due to inappropriate repair of induced DSBs. Scale bars = 5μm.

Much of what we know about HR progression in the germ line is based on cytological analyses of recombination markers in mutants that perturb different aspects of recombination. While probes for different steps of recombination continue to be developed, widely used markers include antibodies against RAD-51, which marks processed DSBs (Alpi *et al.* 2003; Colaiácovo *et al.* 2003) (Figure  4), and fluorescent fusions of the meiosis-specific COSA-1/CNTD1 (cyclin-related; CrossOver Site Associated 1) protein (Figure  5A) or the predicted SUMO-ligase ZHP-3 (Zip Homologous Protein 3), which mark the six CO designation sites, one on each of the six chromosome pairs that make up the *C. elegans* diploid genome (Bhalla *et al.* 2008; Yokoo *et al.* 2012). Additionally, in diakinesis, the six chromosome pairs are easily observed as six individual cruciform structures, or bivalents, as a result of chiasmata formation. Failure to form a chiasma leads to either univalent chromosomes (*i.e.*, chromosomes not connected by a CO), or chromatin aggregates if NHEJ, MMEJ, and/or SSA are engaged and thereby generate chromosome fusions (Figure  3B). Thus, the number and shape of DAPI-staining bodies at diakinesis provides a simple readout of the success of meiotic recombination. These properties have made the *C. elegans* hermaphrodite germ line a premier system for investigating mechanisms of meiotic recombination.

The male germ line is arranged similarly to the hermaphrodite germ line, with some differences. One, meiotic progression occurs approximately two times as fast during male spermatogenesis compared to hermaphrodite oogenesis, and germ cells progress through the different meiotic prophase stages with different kinetics in the two sexes (Jaramillo-Lambert *et al.* 2007; Shakes *et al.* 2009). Two, due to tight packing of the DNA into sperm, chromosomes are not individualized at diakinesis, as in hermaphrodites, and thus the main assays for HR progression rely on cytological analyses of markers (Figures  4 and 5). Three, the meiotic divisions occur within the male gonad, while in hermaphrodites, the meiotic divisions occur following fertilization. Interestingly, several aspects of meiotic recombination and checkpoint signaling are differently regulated during male *vs* female meiosis (Jaramillo-Lambert *et al.* 2010; Checchi *et al.* 2014).

### Challenges to genome integrity during development

Embryogenesis occurs over a period of 8 h, and ∼2/3 of the 1090 cells generated during the largely invariant somatic development are born during this period. Embryonic cell cycles are very fast, with the first cell divisions lasting ∼20 min (Sulston *et al.* 1983). Maintaining genome stability during these rapid cell divisions, largely consisting of alternating S and M phases, is a major challenge. The relative contributions of the various DSB repair and recombination pathways operating during embryogenesis have not yet been systematically analyzed, but NHEJ appears to have an important role (Clejan *et al.* 2006). The finding that NHEJ, together with TLS, have major roles in DNA repair in embryos suggests that the requirement for rapid embryonic cell divisions has selected for these fast acting, error-prone modalities (Kim and Michael 2008; Roerink *et al.* 2012). In line with this view, cell cycle checkpoints have only a minor role during embryonic cycles, and checkpoint-induced apoptosis does not occur during embryogenesis (Gartner *et al.* 2000; Brauchle *et al.* 2003). The death of the 131 cells that die by developmental apoptosis, largely during embryogenesis but also in the early stages of larval development, are not related to checkpoint regulation (Sulston *et al.* 1983; Gartner *et al.* 2000). Cellular divisions are largely limited to embryogenesis; however, during larval development neuronal tissues and lineages related to vulva development continue to divide (O’Connell *et al.* 1998). Although few divisions occur outside of embryogenesis, cells continue to grow, necessitating a molt at each larval stage, where a new exoskeleton is laid down.

A single germline precursor cell is specified in the first embryonic cell division, and after three rounds of division where the posterior daughter maintains the germ cell fate, two germ cells, termed Z2 and Z3, are set apart, each acting as a founder of one of the two gonads. Germ cell proliferation starts at the L1 stage and continues throughout adulthood to expand to approximately 1000 cells per gonad. Thus, the germ line is the only proliferative tissue in adults (Strome 2005). In the gonad, DSB repair is predominantly mediated by HR. In contrast, NHEJ is the main DSB repair modality in somatic cells (Pontier and Tijsterman 2009) and postreplicative cells of adult worms (Vermezovic *et al.* 2012).

In the L1 stage, the first germ cell division is particularly challenging as each of the two germ cell founders only divides after a protracted period of quiescence (Butuči *et al.* 2015; Wong *et al.* 2018). Resumption of cell division coincides with zygotic gene activation; no gene expression occurs in embryonic germ cells. In this developmental setting, the massive onset of global transcription leads to conflicts with DNA replication, resulting in the formation of R-loops, stable three-stranded nucleic acid structures where an RNA molecule binds to one DNA strand, leaving the second strand single-stranded. Such structures are prone to DNA breakage, most likely caused by topoisomerase II-mediated cleavage, and lead to checkpoint activation (Butuči *et al.* 2015). Indeed, topoisomerase II (TOP-2)-mediated DSBs are required for the loading of the chromatin decompaction factor RUVB-1/2, needed for global chromatin decompaction (Wong *et al.* 2018).

Excessive R-loop formation leading to DNA breakage and DNA damage checkpoint activation also occurs when repetitive and transposon-containing heterochromatic regions of the genome are derepressed (Zeller *et al.* 2016; McMurchy *et al.* 2017). H3K9 histone methylation is one of the repressive chromatin marks; MET-2 is a homolog of mammalian SETDB1 methyltransferase and is required for H3K9 mono and dimethylation, while SET-25, a protein related to mammalian SUV39h1, SUV39h2, and G9a enzymes, mediates H3K9 trimethylation. In *met-2; set-25* double mutants there is excessive transcription of otherwise silent, heterochromatic DNA. This leads to R-loop formation, increased mutation rates in heterochromatic transposon or repeat containing DNA, DNA damage checkpoint activation, and increased p53/CEP-1-induced apoptosis (see *DDR, checkpoint signaling, fail-safe mechanisms, and apoptosis induction*) (Zeller *et al.* 2016; McMurchy *et al.* 2017). Removal of both MET-2 and the HR BRC-1-BRD-1 complex also results in synthetic lethality, suggesting that BRC-1-BRD-1 plays a role in resolving R-loops and silencing heterochromatic sequences (Padeken *et al.* 2019). Related phenotypes are also observed in mutants defective for H3K9me-binding proteins HPL-1, HPL-2, and LIN-61 (Johnson *et al.* 2013; McMurchy *et al.* 2017), as well as *prg-1* argonaute mutants defective in 21 nt piRNA generation, or *hrde-1* mutations defective for generating secondary nuclear 22 nt RNAs (McMurchy *et al.* 2017). MET-2 also interacts with the conserved SMCR-1 protein, a SWI/SNF ATPase proposed to protect genome integrity by promoting the repair and restart of stalled DNA replication forks, supporting a direct role for MET-2 in DNA repair. *smcr-1* mutants are hypersensitive to hydroxyurea (HU), a nucleotide analog that perturbs DNA replication, are defective for the DNA replication checkpoint (see *The DNA replication checkpoint*), enhance the defect of *dog-1* mutants (FANCJ helicase; see *Mutational processes associated with DOG-1/FANCJ and TLS polymerase deficiencies*), have elevated levels of R-loops, and become progressively sterile. MET-2 nuclear accumulation is increased upon DNA replication stress and this increase is partially dependent on SMRC-1, suggesting that SMRC-1-dependent recruitment of MET-2 to the nucleus may serve a function at the replication fork. *met-2; smcr-1* double mutants succumb to sterility earlier than either single mutant lines, and this may be the cumulative effect of severely reduced H3K9 methylation in combination with DNA damage (Yang *et al.* 2019). Progressive sterility is also observed in strains defective for the SET-2 H3K4 methyltransferase. In addition, *set-2* homozygous mutants are hypersensitive to IR and methyl methansulfonate (MMS) treatment, more so in late generations, suggesting that chromatin state plays a critical role in maintaining genome integrity during embryogenesis (Herbette *et al.* 2017).

In summary, the DDR is particularly challenged when cells rapidly divide such as in the early embryo. The DDR is also critical when cells naturally induce DNA DSBs, which occurs during *C. elegans* zygotic gene activation in germ cell primordia, during meiosis (see below), and when excessive transcription conflicts with DNA replication. Consequently, DNA repair modalities and checkpoint signaling play key roles at these critical developmental transitions.

## DNA repair

*C. elegans* contains all of the major pathways to correct altered bases and has been a useful system to uncover the underlying network of proteins important for repair. Here, we summarize the major repair modalities in *C. elegans* and compare and contrast with studies in mammals and other organisms.

### Direct repair

DNA repair by direct reversal eliminates DNA or RNA modifications without the excision of base(s), repair synthesis, or ligation. These reactions are conducted by two major classes of proteins, O6-methyl‐guanine-DNA methyltransferases (MGMT) and ALKBH α-ketoglutarate Fe(II) dioxygenases (Schärer 2012; Ahmad *et al.* 2015). In mammals, there is a single MGMT and nine ALKBH orthologs, four of which have been confirmed to remove alkyl damage in DNA (Aravind and Koonin 2001). To date, two members of MGMTs, AGT-1 and AGT-2 alky guanyl transferases, but no ALKBH orthologs, have been identified in *C. elegans*. AGT-1 is the true ortholog of MGMT and has been shown to repair O6- methyl-guanine *in vitro* (Kanugula and Pegg 2001). Consistent with a role in removing the O6-methyl group from guanine, *agt-1* mutants are associated with increased C > T mutation upon MMS treatment (Volkova *et al.* 2020). In contrast, although AGT-2 has DNA akyltransferase activity *in vitro*, the *in vivo* specificity is not known. *agt-2* mutants show increased apoptosis upon treatment with IR or the methylating agent methylnitronitrosoguanidine. In addition, even in the absence of exogenous damaging agents, *agt-2* mutants have reduced embryonic viability, likely due to a partial impairment of meiotic DSB repair (Serpe *et al.* 2019). When grown over many generations, *agt-2* strains show an approximately twofold increased rate of point mutations, many of which are clustered (Meier *et al.* 2021). The decreased diversity of enzymes involved in direct reversal in *C. elegans* compared to mammals may reflect a lower propensity of spontaneous DNA alkylation in worms.

### BER, hydrolysis of oxidized nucleotides by MutT homologs, and SSBR

The BER pathway involves the action of glycosylase enzymes that recognize and excise a variety of damaged bases through the hydrolysis of the *N*-glycosylic bond between the modified base and deoxyribose, leaving an abasic site with an intact phospho-ribose backbone (Krokan and Bjørås 2013; Beard *et al.* 2019). Only two such enzymes [in contrast to 5 in *Saccharomyces cerevisiae* (Skoneczna *et al.* 2018) and 12 in humans (Krokan and Bjørås 2013; Beard *et al.* 2019)], UNG-1 and NTH-1, have been described in *C. elegans*, likely because glycosylase enzymes have been lost in the nematode lineage, and/or because *C. elegans* enzymes are highly diverged. UNG-1 removes uracil (Nakamura *et al.* 2008; Skjeldam *et al.* 2010), while NTH-1 removes 5-hydroxymethyluracil (5-hmU), an oxidation product of thymine, and other bases (Papaluca *et al.* 2018). Genome analyses indicate an increased number of C > T mutations in *ung-1* mutants, likely caused by uracil–adenine pairing when UNG-1 fails to eliminate uracil that arises from misincorporation or spontaneous cytosine deamination (Meier *et al.* 2014; Volkova *et al.* 2020). Following uracil removal, APN-1 and EXO-3 act redundantly to incise the resulting apurinic/apyrimidinic (AP) sites and remove 3′-blocking lesions at DNA SSBs (Yang *et al.* 2012; Papaluca *et al.* 2018). Interestingly, when uracil misincorporation into DNA is increased by depleting the *C. elegans* dUTP nucleotide hydrolase DUT-1, both RPA-1 and checkpoint kinase ATL-1 are recruited to chromatin, leading to the activation of the DNA damage checkpoint pathway, which results in cell cycle arrest and apoptosis induction (Dengg *et al.* 2006). Checkpoint activation requires UNG-1-dependent uracil excision. The suppression of the lethality conferred by DUT-1 depletion in *clk-2* checkpoint mutants, which are defective for the DDR and S-phase checkpoint (Ahmed *et al.* 2001), suggests that excessive uracil excision leads to lethality caused by checkpoint hyperactivation.

To avoid base damage, additional mechanisms exist that remove oxidized nucleotide precursors so that they cannot be incorporated into DNA during replication. Bacterial MutT and its homologs function by cleaving phosphate groups from oxidized nucleotide precursors (Lu *et al.* 2001). Three MutT homologs (NDX-1, NDX-2, and NDX-4) have been characterized in *C. elegans.* NDX-1 cleaves 8-oxo-dGDP but not 8-oxo-dGTP (Sanada *et al.* 2011), while NDX-4 cleaves 8-oxo-dGTP but not 8-oxo-dGDP (Arczewska *et al.* 2011), and NDX-2 is the hydrolase for 8-oxo-dGDP (Sanada and Zhang-Akiyama 2014). *C.* *elegans* MutT mutant strains are hypersensitive to oxidative damage, consistent with a role in eliminating oxidized bases.

SSBs generated by BER or directly by DNA damage are repaired by the SSBR pathway, which has been understudied in *C. elegans*. A key step of SSBR is the recruitment of PAR polymerases (PARPs) to SSBs and their autocatalytic polyADP‐ribosylation (PARylation). PARylated PARP serves as a recruitment platform for DNA repair enzymes. While there are 17 PARP enzymes in mammals, with PARP1, PARP2, and PARP3 having well-characterized roles in DNA repair, only PARP-1 and PARP-2 (formally PMS-1 and PMS-2) are encoded in *C. elegans* (Gagnon *et al.* 2002; Dequen *et al.* 2005b). PARP-1 and PARP-2 have PARylation activity and mutations are associated with increased sensitivity to IR (Dequen *et al.* 2005b). PAR glycohydrolase (PARG) enzymes remove PAR chains and are associated with PARP1 turnover needed for the completion of DNA repair. *C.* *elegans* has two paralogs of PARG, PARG‐1 and PARG‐2, previously referred to as PME‐3 and PME‐4, and deletion of either causes hypersensitivity to IR (St-Laurent *et al.* 2007; Bae *et al.* 2020). RPA-1 foci appear precociously in *parg-2* mutants, a phenotype suppressed by exonuclease (*exo-1*) mutants (Bae *et al.* 2020). Interestingly, PARG-1 has recently been shown to function in meiotic DSB formation and repair independently of its catalytic activity (Janisiw *et al.* 2020).

In conclusion, as in other organisms, *C. elegans* has multiple pathways to detect, remove, and repair damaged bases both prior to and following incorporation into the DNA double helix. The complexity of these pathways appears to be reduced in worms, likely due to the streamlined nature of the genome.

### DNA mismatch repair

The major role of DNA MMR is to correct replication errors conferred by replicative polymerases. Replication errors are recognized and detected by two MutS complexes, MutSα and MutSβ, comprised of MSH2/MSH6 and MSH2/MSH3 heterodimers (Drummond *et al.* 1995; Habraken *et al.* 1996; Genschel *et al.* 1998). In contrast, the meiosis-specific MutSγ complex, a heterodimer of MSH4 and MSH5, does not function in mismatch correction but is essential for CO formation in *C elegans* as in other organisms (Kelly *et al.* 2000; Colaiácovo *et al.* 2003) (see *DSB repair*). With respect to MMR, MutS binding to the mismatch facilitates the recruitment of the MutL complex (MutH in *E. coli*). MutL enhances mismatch recognition and triggers a conformational change in MutS, leading to the sliding of the MutL/MutS complex away from the mismatched DNA (Allen *et al.* 1997; Gradia *et al.* 1999). DNA repair is initiated by a single-stranded nick generated by MutL on the nascent DNA strand (Kadyrov *et al.* 2006, 2007). Exonucleolytic activities, in part conferred by the EXO1 nuclease, contribute to the removal of a short stretch of DNA containing the mismatch. This is followed by gap filling *via* lagging strand DNA synthesis (Goellner *et al.* 2015). Out of the three MutLα, MutLβ and MutLγ complexes encoded in the mammalian genome, only MutLα subunits MLH-1 and PMS-2 can be identified in the *C. elegans* genome (Degtyareva *et al.* 2002; Tijsterman *et al.* 2002). MMR deficiency leads to enhanced mutagenesis as measured by *unc-58/unc-93* reversion assays, and by using lacZ reporter constructs that allow for the restoration of a reading frame in an homopolymeric stretch of DNA (Degtyareva *et al.* 2002; Tijsterman *et al.* 2002). Amongst all DNA repair mutants, spontaneous mutation rates are highest in MMR mutants, causing pogressive sterility when homozygous lines are propagated over many generations (Denver *et al.* 2005; Meier *et al.* 2018; Volkova *et al.* 2020). In addition to a mutator phenotype, MMR deficiency in humans is characterized by instability of DNA repeat sequences (microsatellite instability). To identify new genes involved in MMR, a genome-wide RNAi screen for microsatellite instability was conducted in *C. elegans* (Tijsterman *et al.* 2002; Pothof *et al.* 2003). The screen identified several genes with known roles in DNA repair and replication, chromatin organization and remodeling, cell cycle and checkpoint control as well as genes with unknown function, suggesting that the complete set of repair genes has not yet been discovered (Tijsterman *et al.* 2002; Pothof *et al.* 2003).

In addition to misincorporation of endogenous nucleotides, the chemotherapeutic agent 5-Fluorouracil (5-FU) leads to elevated levels of nucleotide misincorporation. 5-FU is metabolically converted to 5-fluoro-2′-deoxyuridine monophosphate, which acts by inhibiting thymidylate synthase, resulting in perturbation of nucleotide pools and the incorporation of dUTP and 5-fluoro-2′-dUTP into DNA (Longley *et al.* 2003). It has been proposed that the incorporation of these nucleotides does not directly lead to the formation of strand breaks, rather DNA breakage is likely generated by repair enzymes (Longley *et al.* 2003; Wyatt and Wilson 2009). This hypothesis is supported by the finding that *C. elegans* MMR and BER deficiency suppress the excessive induction of autophagy and the reduced progeny viability resulting from 5-FU exposure (SenGupta *et al.* 2013). In line with MMR and BER producing toxic intermediates, the DNA damage checkpoint activation triggered by 5-FU exposure is also suppressed in MMR and BER mutants (SenGupta *et al.* 2013). These studies highlight the interconnections between DNA repair modalities and checkpoint signaling, and the advantages of *C. elegans* to uncover mechanisms underlying the *in vivo* toxicity of 5-FU and other cancer chemotherapies.

### Nucleotide excision repair

NER acts by detecting a distortion of the double helix formed by bulky adducts or interlinked bases, and by excising a ∼30 bp stretch of ssDNA carrying the damaged base(s) (Schärer 2013) (Figure  1A). NER repairs lesions that arise throughout *C. elegans* development (Astin *et al.* 2008; Lans *et al.* 2010; Ermolaeva *et al.* 2013). Transcriptionally quiescent dauer larvae are resistant to acute UV treatment, but UV-treated dauer larvae fail to develop into adults upon the addition of food, suggesting that there is a complicated relationship between food sensing and NER (Astin *et al.* 2008). NER, which is conserved between mammals, yeast, and *C. elegans*, is organized into two branches. Global genome repair (GG)-NER scans the entire genome, while transcription-coupled (TC)-NER recognizes lesions at sites of RNA polymerase II stalling (Schärer 2013) (Figure  1A). In humans, congenital mutations affecting global genome NER (GG-NER) result in Xeroderma pigmentosum, one of the first inherited diseases associated with a DNA repair deficiency, characterized by exquisite sensitivity to UV irradiation and a >1000-fold increased risk of skin cancer (Black 2016). In contrast, patients defective for transcription coupled nucleotide excision repair (TC-NER) deficiency suffer from Cockayne syndrome, characterized by growth retardation, cachexia, neuronal degeneration, progeric appearance, and premature death without excessive hypersensitivity to UV (Marteijn *et al.* 2014; Lans *et al.* 2019).

In *C. elegans*, as in other organisms, GG-NER and TC-NER differ only in the initial steps of DNA damage recognition. The GG-NER pathway employs DNA-damage binding (DDB-1) and the XPC-1-RAD-23 complex to scan the genome and recognize helix distortions (Lans and Vermeulen 2011) (Figure  1A). Additionally, XPA-1 performs a function in damage recognition that is, as yet, poorly defined. In contrast, TC-NER is initiated when RNA polymerase II stalls at a DNA lesion. This requires the binding of Cockayne Syndrome proteins CSA-1 and CSB-1. CSA-1 is a WD-40 repeat protein scaffold protein and CSB-1 is a SWI2/SNF-2 family type chromatin remodeler (Babu *et al.* 2014; Lopes *et al.* 2020) (Figure  1A). Upon identification of a damaged site by these different sensors, the same repair proteins are recruited to excise the damaged DNA surrounding the lesion and to then fill in the repair patch during both GG-NER and TC-NER. 5′ incision is carried out by ERRC-1/XPC-1, while the 3′ incision is mediated by the XPG-1 nuclease (Lans and Vermeulen 2011) (Figure  1A). Genetic analyses are consistent with this view as mutations in shared components of these two pathways show the strongest sensitivity to UV irradiation (Schärer 2013).

Interestingly, GG-NER and TC-NER are differentially used during development (Marteijn *et al.* 2014; Mueller *et al.* 2014; Borgermann et al. 2019; Lans *et al.* 2019; Lopes *et al.* 2020; Sabatella *et al.* 2021). GG-NER is the predominant pathway used in the germ line, and corresponding mutations lead to reduced progeny viability of UV-treated worms. GG-NER is particularly efficient in late stage oocytes (Sabatella *et al.* 2021). In contrast, TC-NER is mainly required during embryonic development. TC-NER deficiency leads to developmental retardation and arrest after exposure to low doses of UV irradiation. The reduction of transcription mediated by UV irradiation is associated with reduced RNA polymerase II levels, as stalled RNA polymerase II is ubiquitinated and degraded by the proteasome (Astin *et al.* 2008). Indeed, levels of the large subunit of RNA polymerase II, AMA-1, are reduced upon UV treatment, and such reduction is blocked upon depletion of the WWP-1 E3 ubiquitin ligase, which is related to budding yeast Rsp5 that has a similar role (Astin *et al.* 2008). When AMA-1 fails to be degraded in *wwp-1* mutants, *C. elegans* becomes more sensitive to UV irradiation, consistent with a requirement for degradation of stalled RNA polymerase II for transcription to resume (Astin *et al.* 2008). In addition to development, TC-NER is required for preventing neurodegeneration; aging *csb-1* animals show mitochondrial dysfunction and neuronal loss that is enhanced upon UV treatment (Lopes *et al.* 2020; Sabatella *et al.* 2021). In postmitotic muscle and neuronal cells, repair mainly occurs in actively transcribed genes; neurons are more susceptible to UV damage compared to muscle cells (Sabatella *et al.* 2021). Consistent with this, *C. elegans csb-1* animals mimic neurodegeneration phenotypes observed in Cockayne syndrome patients, which is not recapitulated in mouse models (Lopes *et al.* 2020). Thus, *C. elegans* is an excellent system to probe the molecular underpinnings of Cockayne syndrome pathology.

Recent evidence indicates that UV repair and the resumption of transcription are interconnected *via* H3K4 methylation, which is associated with active transcription (Wang *et al.* 2020b). A basal level of H3K4me2 is required for efficient DNA repair, as measured by the kinetics of cyclobutane pyrimidine dimer removal upon UV irradiation. In addition, H3K4me2 is induced upon UV treatment, and this induction requires active NER. Based on these findings it was proposed that NER-dependent H3K4me2 deposition ensures that transcription can fully resume once DNA repair is completed. Consistent with this model, the WRAD SET-1 H3K4 methyltransferase complex, together with the SET-2 and MLL1-like SET-16 H3K4 methyltransferase-containing MLL/COMPASS complex, are required for efficient repair of UV damage, and mutations in *wdr-5* and *ash-2*, noncatalytic WRAD complex components, are UV hypersensitive. Conversely, deletions of the SPR-5 and AMX-1 H3K4me2 demethylases confer UV resistance (Wang *et al.* 2020b). Thus, H3K4me2 couples NER with transcription. Interestingly, *spr-5* demethylase defective worms are also hypersensitive to DNA replication stress and IR, have persistent meiotic DSBs, and are partially compromised for checkpoint activation in response to stalled replication forks, suggesting a general role for modulation of H3K4 methylation state in DNA damage repair and signaling (Nottke *et al.* 2011; Kim *et al.* 2018). There is also evidence that the FA pathway (see below) is required for maintaining low H3K4me2 levels, and that the FA FNCM-1 helicase directly interacts with the SPR-5 demethylase (Kim *et al.* 2018).

The developmental defects associated with transcription-coupled repair are enhanced in short-lived mutants defective for the conserved DAF-16 FOXO transcription factor, which acts downstream of insulin signaling. When insulin signaling is compromised, DAF-16 is derepressed resulting in partial resistance to UV damage (Edifizi and Schumacher 2015; Tissenbaum 2018). DAF-16 is required to confer resistance to a large variety of stresses, while the EGL-27/GATA transcription factor, in conjunction with DAF-16, appears to antagonize the effects of UV damage (Mueller *et al.* 2014). It is not clear if DAF-16 and EGL-27 directly increase TC-NER repair capacity, or if they act by antagonizing organismal stress response pathways activated by excessive DNA damage and/or by perturbed transcription. Interestingly, the UV DDR and *C. elegans* organismal stress responses are interlinked (Ermolaeva *et al.* 2013) (see *Links between DNA damage signaling and organismal stress responses*). Structure–function analyses using a *daf-16* allele, lacking the transcriptional transactivation domain, provides evidence that the role of DAF-16 in protecting against UV damage is independent of transcription, possibly related to binding RPA-1 (Daitoku *et al.* 2016).

TC-NER mutants show only marginally increased mutagenesis when propagated over many generations (Volkova *et al.* 2020). It will be interesting to determine if mutagenesis in somatic tissues is increased to an extent that can explain the neurodegeneration phenotypes in TC-NER mutants. NER is required to repair ∼90% of all base changes and short insertion deletions caused by the bulky DNA adduct aristolochic acid and UV irradiation (Volkova *et al.* 2020). A smaller proportion of ∼30–60% base changes caused by alkylating agents dimethyl methanesulfonate (DMS), ethyl methanesulfonate (EMS), and methyl methanesulfonate (MMS) are prevented by NER, the smaller effect likely being due to methylation and ethylation leading to a lesser extent of helix distortion (Volkova *et al.* 2020) (for details, see *Using C. elegans to define mutational signatures*).

All in all, the NER pathway is well conserved in *C. elegans*. The worm system provides an ideal model to study the differential use of NER in various tissues, and the linkage of NER defects with neurodegeneration and aging.

### Interstrand and Protein CLR

DNA ICLs are one of the most toxic DNA lesions and are challenging to repair, especially in the context of DNA replication. DNA crosslinks are caused by endogenous metabolites such as the lipid peroxidation product malondialdehyde, or acetaldehyde, a major metabolite of alcohol, and lead to guanine-to-guanine interstrand crosslinkage [for review, see Rogers *et al.* (2020)]. In addition, abasic sites resulting from the action of BER lead to crosslinks through an aldehyde intermediate. In clinical settings, DNA crosslinking agents such as cisplatin and mitomycin-C are used as cytotoxic agents for cancer treatment, and the combined exposure to UV and trimethylpsoralen (UV–TMP) is used to treat excessive skin cell growth in psoriasis (Rogers *et al.* 2020).

ICL repair requires several steps (Figure  1, B and C). ICLs first must be recognized, and in a process referred to as “unhooking,” an ssDNA stretch bound to the ICL is excised by structure-specific nucleases. During S-phase, DNA replication converts the single-stranded gap into two DSBs, which are repaired by HR. Error prone translesion polymerases read across the remaining adduct on the other strand (Deans and West 2011; Ceccaldi *et al.* 2016; Hashimoto *et al.* 2016). In mammalian cells ICL repair is largely mediated by the FA pathway, ICL repair being linked to DNA replication with one or two replication forks converging at a persistent ICL (Figure  1B).

FA is a congenital disease characterized by short stature, developmental disabilities, bone marrow failure, increased cancer incidence, hypersensitivity to DNA crosslinking agents, and complex chromosomal aberrations (Ceccaldi *et al.* 2016). When replication forks collide with an ICL, the replicative MCM2-7 helicase is displaced by the BRCA1-BARD1 complex, thereby facilitating the activation of the FA pathway (Fu *et al.* 2011; Wu *et al.* 2019). ICLs are then recognized by the FA FANCM complex, which functions as a landing platform for the 14 subunit FA core complex, a multisubunit E3 ubiquitin ligase, which acts with the UBE2T E2 ubiquitin-conjugating enzyme to facilitate the monoubiquitylation of FANCD2 and FANCI (Ceccaldi *et al.* 2016). While those ubiquitylation events were considered to be required for the recruitment of downstream repair factors, more recent structural and biochemical studies indicate that monoubiquitylation facilitates the formation of a ring-like structure composed of FANCD2 and FANCI that stably encircles double-stranded DNA (dsDNA) and possibly acts as a sliding clamp to recruit other repair factors, protect DNA, and/or serve as a processivity factor (Niraj *et al.* 2019; Shakeel *et al.* 2019; Wang *et al.* 2020a; Rennie *et al.* 2020). Other components of the FA pathway, mutations of which are all associated with *bona fide* congenital FA, including BRCA2 and its binding partner FANCN, the FANCJ helicase (DOG-1 in *C. elegans*) as well as the XPF1 nuclease and the SLX4 multinuclease-scaffold protein (which provides a scaffold for XPF1, MUS81, and SLX1 nucleases), appear to function in downstream repair roles, as they are not required for the monoubiquitylation of FANCD2 and FANCI [for review, see Ceccaldi *et al.* (2016)]. Other proteins, including the SMN1A and FAN1 nucleases, as well as the HELQ helicase, are needed for ICL repair without having a clear role in the canonical FA pathway.

*C.* *elegans* has five core FA proteins, BRC-2, FCD-2, FNCI-1, DOG-1/FANCJ, and FNCM-1, in addition to the XPF-1 nuclease, SLX-4, and RAD-51 paralogs. Components of the large E3 ligase complex have not been identified in the nematode, although it is possible that homology searches have failed due to a lack of sequence conservation (Dequen *et al.* 2005a; Collis *et al.* 2006; Youds *et al.* 2009). Mutants defective for the five core FA proteins are hypersensitive to UV–TMP and cisplatin, consistent with a role in ICL repair (Dequen *et al.* 2005a; Collis *et al.* 2006; Lee *et al.* 2007). The loading of FCD-2/FANCD2 in response to UV–TMP requires FNCM-1, FNCI-1, and RPA-1, as well as the ATL-1-CHK-1 DNA damage checkpoint kinase cascade (Lee *et al.* 2010b). As in mammalian cells, FCD-2 also forms chromatin-associated foci in response to DNA replication stress, but FCD-2 is not required for the activation of the S-phase checkpoint (Collis *et al.* 2006). Although *dog-1* mutants are hypersensitive to ICL treatment, it is mechanistically unclear how DOG-1/FANCJ contributes to DNA CLR. On the other hand, studies in *C. elegans* were instrumental in showing that this helicase is required for replication through G-quadruplexes (G4) forming DNA structures (Wu *et al.* 2008; Kruisselbrink *et al.* 2008) (discussed in *Using C. elegans to define mutational signatures*).

Intriguingly, NHEJ contributes to the hypersensitivity of FA-defective worms to crosslinking agents. This phenotype appears to be caused by inappropriate ligation of chromatin breaks that occur during ICL repair by the FA pathway (Adamo *et al.* 2010; Pace *et al.* 2010). Indeed, chromosomal aberrations, which include fusions between homologous and heterologous chromatids, a cytological hallmark of FA cells, appear to be largely caused by end joining activity, suggesting that blockage of DNA end-joining might be beneficial for patients suffering from FA.

A recent systematic analysis of *C. elegans* mutants defective for FA, NER, BER, and HR pathways revealed that NER mutants are much more sensitive to UV–TMP compared to FA mutants, suggesting that NER plays a prominent role in repairing ICLs in *C. elegans* (Wilson *et al.* 2017). Given that the XPF-1/FANCQ nuclease is involved in the NER excision step and in unhooking in the FA pathway, the hypersensitivity of *xpf-1* mutants was not unexpected. However, mutants of genes known to solely affect NER, such as the XPG-1 nuclease and the XPA-1 damage sensor, clearly demonstrate that the NER pathway is used to repair ICLs in *C. elegan*s (Wilson *et al.* 2017). Furthermore, both GG-NER and TC-NER appear to be involved as *xpc-1; csb-1* double mutants show increased ICL sensitivity compared to the corresponding single mutants. In addition, *C. elegan*s REV-1 and POL ζ translesion polymerases appear to act redundantly in ICL repair (Oh *et al.* 2020), and double mutants defective for either of those polymerases and NER are exquisitely sensitive to DNA crosslinks induced by UV–TMP treatment (Oh *et al.* 2020). Together, these data suggest that ICLs in *C. elegans* are largely repaired by a mechanism independent of the FA pathway, most likely through NER. UV–TMP hypersensitivity is observed in NER mutants during larval and germline development, stages where excessive proliferation occurs and the proportion of S-phase cells is high (Crittenden *et al.* 2006; Fox *et al.* 2011). NER may also repair ICLs outside of S-phase as young adult NER-defective worms treated with UV–TMP have reduced pharyngeal pumping rates due to death, or reduced survival of neurons and muscle cells (all somatic cells in adults are postmitotic). Thus, the FA pathway appears to only have a minor role in *C. elegans*, which, like *Dictyostelium*, *Drosophila* and yeast, encodes for a restricted set of FA proteins (McVey 2010).

Given the prominent role of the NER pathway in *C. elegans* ICL repair, it has been difficult to determine if mutants defective for ICL repair act in the FA pathway, especially when FCD-2 focus formation is unaffected. Several factors seem to be involved in the *C. elegans* FA pathway downstream of FCD-2. The RNF-113 ring finger protein appears to be epistatic to FCD-2, acting downstream of ICL-induced focus formation, and is likely involved in HR, as RPA-1 foci persist while RAD-51 foci are reduced in *rnf-113* mutants (Lee *et al.* 2013). Similarly, mutants defective for the *C. elegans* JMJD-1.1/PHF8 (KDM7A) JmjC domain-containing H3 mono- and dimethyl K9 demethylase acts downstream of FCD-2 loading, likely affecting HR at a step after assembly of the RAD-51 recombinase (Lee *et al.* 2015). The FAN1 structure-specific nuclease has also been proposed to act downstream of FANCD2 as a target of FANCD2 ubiquitinylation (Kratz *et al.* 2010; MacKay *et al.* 2010; Smogorzewska *et al.* 2010); however, more recent data suggest that FANCD2-dependent FAN1 targeting is required for the ICL-independent role of the FA pathway in slowing down replication forks upon DNA replication stress (Lachaud *et al.* 2016a). Moreover, the role of FAN1 in ICL repair does not require targeting by FANCD2, and mutation of FAN1 does not result in FA but is associated with karyomegalic interstitial nephritis, a chronic kidney disease (Zhou *et al.* 2012; Lachaud *et al.* 2016b). Finally, FAN1 knockout mice are sensitive to ICLs; however, FANCD2 FAN1 double mutants are more sensitive than each single mutant, consistent with FANCD2 and FAN1 functioning in two independent pathways (Lachaud *et al.* 2016a). *C.* *elegans fan-1* mutants are also more sensitive to ICLs compared to *fcd-2*, consistent with FAN-1 and FCD-2 functioning in different pathways (Kratz *et al.* 2010; MacKay *et al.* 2010; Smogorzewska *et al.* 2010). Interestingly, *C. elegans* FAN-1 localization at repair foci depends on the *C. elegans* SUN domain protein UNC-84 (Lawrence *et al.* 2016). *C.* *elegans* UNC-84 functions in nuclear migration; UNC-84 spans the nuclear periphery through the inner and outer nuclear membrane in complex with the KASH domain protein UNC-83, which in turn connects to the cytoskeleton, as part of the conserved LINC complex. Mechanotransduction by the *C.elegans* SUN-1-ZYG-12 LINC complex has an important role in the early stages of meiotic chromosome pairing (Malone *et al.* 2003; Penkner *et al.* 2009), and mammalian UNC84 plays a role in driving the microtubule-dependent inhibition of NHEJ by binding to the KU70/KU80/LIG-4 DNA-PK machinery (Lottersberger *et al.* 2015)*. unc-84* and *zyg-12* mutants are hypersensitive to ICL agents, and consistent with a downstream role in ICL repair, RAD-51 foci numbers are initially decreased after ICL treatment, but later increase compared to wild type. An initial decrease in RAD-51 foci is also observed when worms are treated with the microtubule poisons colchicine and nocodazole, consistent with MT movement and dynamics being required for efficient ICL repair. As is the case for *fcd-2* mutants (Adamo *et al.* 2010; Pace *et al.* 2010), the hypersensitivity of *unc-84* mutants is suppressed by NHEJ deficiency (in nematodes and human cells), suggesting that inappropriate ligation of chromatin breaks might lead to complex chromosomal aberrations and fusions, resulting in lethality.

MRT-1 is another nuclease involved in *C. elegan*s ICL repair (Meier *et al.* 2009). It is most related to SNM1A, which is one of the three metallo‐β‐lactamase fold and β‐CASP containing nucleases encoded in the human genome, and the functional homolog of budding yeast PSO1 [for review, see Baddock *et al.* (2020)]. Snm1A has been proposed to remove crosslinks through its exo-nucleolytic 5′–3′ activity, following incision 5′ of the DNA crosslink by the XPF1 nuclease (Wang et al. 2011). It is likely that mechanisms redundant with such nuclease activity exist as mutations in Snm1A are not associated with FA (Baddock *et al.* 2020). Interestingly, MRT-1 contains a POT-telomere-binding protein-type OB fold in addition to the Snm1 metallo‐β‐lactamase fold nuclease domain (Meier *et al.* 2009). Consequently, MRT-1 is required for telomere maintenance, and mutations are sensitive to IR and UV–TMP. MRT-1 functions as a 3′–5′ nuclease *in vitro*, but it is not known how it mechanistically acts to mediate telomerase maintenance or ICL repair.

Besides crosslinks between two DNA strands, linking DNA and proteins is equally toxic. Such crosslinks can be mediated by formaldehyde, a major catabolite of ethanol and produced as a byproduct of histone demethylation reactions on chromatin (Nakamura and Nakamura 2020). Protein–DNA adducts can also arise as part of topoisomerase reactions. Topoisomerases are transiently bound to DNA *via* a reactive tyrosine and abortive reactions lead to DNA–topoisomerase crosslinks. Several specialized proteases, such as the DVC-1/SPRTN metalloprotease, remove protein adducts [for reviews, see Dizon and Reinking (2017) and Nakamura and Nakamura (2020)]. *C.* *elegans dvc-1* mutants and SPRTN RNAi-treated human cells are exquisitely sensitive to formaldehyde, without showing increased sensitivity to a variety of unrelated DNA damaging agents (Stingele *et al.* 2016). SPRTN chromatin targeting requires a deubiquitinylation step, and SPRTN protease activity is activated by DNA binding, with SPRTN autodegradation likely part of a negative feedback needed for SPRTN inactivation (Stingele *et al.* 2016). DVC-1/SPRTN acts in parallel with the DVC-1/SPRTN paralog, GCNA-1 (also termed ACRC). GCNA-1 directly interacts with TOP-2 topoisomerase and the corresponding mutant is hypersensitive to TOP-2 poisons (Borgermann et al. 2019; Bhargava *et al.* 2020; Dokshin *et al.* 2020). Human GCNA1 is targeted to damage sites by sumoylation *via* its SIM SUMO-binding domains, and RNAi depletion of SUMO and SUMO ligase also results in cells that are formaldehyde hypersensitive. *gcna-1; dvc-1* double mutants are equally sensitive to formaldehyde as the corresponding single mutants, indicating that *gcna-1* and *dvc-1* act in the same pathway to repair DNA-protein crosslinks (Borgermann et al. 2019). On the other hand, GCNA-1 and DVC-1 appear to have an additive role in preserving genome integrity in untreated meiotic cells (Bhargava *et al.* 2020). *gcna-1* mutants become sterile when propagated over multiple generations, and mutation rates are 10- to 30-fold increased in a genetic reversion assay scoring for suppressors of a movement defective *unc-58* allele, presumably due to abortive topoisomerase reactions (Harris *et al.* 2006; Dokshin *et al.* 2020). Reversions are caused by deletions ranging from 1 to 51 kb, duplications and complex rearrangements (Dokshin *et al.* 2020). Whole genome sequencing provides evidence for mutagenesis within complex multicopy tandem repeat loci (Dokshin *et al.* 2020). In humans, GCNA copy number loss and silencing is particularly evident in pediatric germ cell tumors; tumors with reduced or aboragated GCNA expression show particularly high levels of genome instability (Bhargava *et al.* 2020).

In conclusion, unlike in mammals, NER, likely in combination with TLS, acts as the major modality for ICL repair in *C. elegans.* It is possible that NER is also involved in ICL repair in mammalian cells, but this role is overshadowed by the FA pathway. Recent work on the DVC-1/SPRTN and GCNA-1 proteases has provided important insights into the repair of protein adducts, especially those linked to aborted topoisomerase reactions.

### Translesion synthesis

When error-free modes of DNA repair fail, TLS polymerases serve as a last resort, catalyzing nucleotide extension across a variety of DNA lesions (Vaisman and Woodgate 2017; Yang and Gao 2018). TLS polymerases are also used in ICL repair, reading across the adduct that remains after the crosslink is severed by “unhooking” (Deans and West 2011; Hashimoto *et al.* 2016; Ceccaldi *et al.* 2016) (see above). TLS polymerases are generally error prone, lack proofreading activity, and have a wider catalytic center to allow reading across damaged bases. Amongst eukaryotic Y-family polymerases that mediate TLS, POLη/POLH-1, POLκ/POLK-1, and REV1/REV-1 are conserved in the nematode; no homolog of Polι has been identified. TLS Polζ is a B-family polymerase comprised of the REV3 catalytic subunit and the REV7 regulatory subunit; REV-3 is conserved in the worm (Roerink *et al.* 2012; van Bostelen and Tijsterman 2017; van Bostelen *et al.* 2020). REV1 also plays a noncatalytic role by interacting with other TLS proteins such as POLη, and the regulatory POLζ subunit REV7 (Vaisman and Woodgate 2017; Yang and Gao 2018).

In *C. elegans*, individual translesion polymerases are not required for survival during normal proliferation. Except for *polk-1* mutants, *C. elegans* TLS mutants are UV-sensitive, while *rev-3; polk-1* double mutants are hypersensitive, consistent with a cryptic role of POLK-1 in UV repair. *rev-1* and *rev-3* single and double mutants are equally UV-sensitive, providing genetic evidence that REV-1 is important for Polζ REV-3 activity (Roerink *et al.* 2012; van Bostelen and Tijsterman 2017; van Bostelen *et al.* 2020). In contrast, *rev-1; polh-1* double mutants are hypersensitive compared to the single mutants, suggesting a redundant role. *polh-1* mutants are also sensitive to IR, cisplatin, and MMS, suggesting that POLH-1 acts on a number of different lesions (Roerink *et al.* 2012). Additionally, POLH-1 and POLK-1 have important roles during the rapid embryonic cell cycles (Kim and Michael 2008; Roerink *et al.* 2012). POLH-1 appears to be regulated by SUMOylation and ubiquitin-mediated degradation by the CUL4-DDB1-CDT2 pathway (Kim and Michael 2008). It was proposed that *C. elegans* GEI-17 SUMO E3 ligase protects POLH-1 from degradation by the CUL4-DDB1-CDT2 pathway until it has performed its function in TLS.

When propagated over multiple generations *rev-1*, *rev-3*, and *polh-1* show an increased rate of 50–400 bp deletions (Volkova *et al.* 2020). These data suggest that REV-3, and perhaps POLH-1, prevent DNA breaks by reading across damaged bases. Resulting breaks are likely repaired by POLQ-1 mediated end joining (van Bostelen *et al.* 2020). *polk-1* mutants show a 50-fold increase of mutagenesis upon EMS, MMS, and DMS treatment (Volkova *et al.* 2020). In contrast, *rev-3*, and to some extent *polh-1* mutants, shows reduced mutations upon UV treatment or exposure to EMS, MMS, aflatoxin, and aristolochic acid (Volkova *et al.* 2020) (for details, see *Mutational processes associated with DOG-1/FANCJ and TLS polymerase deficiencies*).

Translesion polymerases have a major role in shaping the genome in response to DNA damage, *polk-1* mutants showing a ∼50-fold increase in single base changes. Reduced levels of base changes observed in *rev-3*, and especially in *polh-1* backgrounds, come at the price of an increased burden of indels and structural variant (SV), likely explaining why these strains are exquisitely sensitive to a variety of DNA damaging agents.

## DSB repair

DSBs arise due to chemical or physical insults, during DNA replication, and are intentionally induced during meiosis. In G1 of the cell cycle and in somatic cells, DSBs are primarily repaired by NHEJ, in which the broken DNA ends are directly religated. NHEJ is considered moderately error prone, as small deletions (1–4 bp) can be generated. In *C. elegans*, as in other systems, the KU70/80 (CKU-70-CKU-80) heterodimer binds and protects the broken ends and recruits a specialized ligase, ligase IV/LIG-4, which catalyzes a phosphodiester bond between the two ends (Clejan *et al.* 2006; Chang *et al.* 2017) (Figure  2). In a number of organisms, ligase IV forms a complex with XRCC4, and NHEJ also requires DNA-PK and the Artemis nuclease; however, no homologs of these proteins have been identified in *C. elegans*. Recently, a new *C. elegans* NHEJ accessory protein, NHJ-1, was identified that appears to function with LIG-4 (Vujin *et al.* 2020). NHJ-1 has no sequence homology outside the Rhadbitid family.

MMEJ is an alternative DSB repair pathway that results in insertions and deletions, and is thus considered error prone. During MMEJ, broken ends are resected to reveal short stretches of homology. Resection is likely mediated by the MRE-11 (Chin and Villeneuve 2001), RAD-50 (Hayashi *et al.* 2007), NBS-1 (Girard *et al.* 2018) [MRE11/RAD50/NBS1 (MRN)] complex (Truong *et al.* 2013), as it is during HR (see below). Polymerase theta promotes annealing of the resulting microhomologies through its helicase activity, while its polymerase activity is responsible for gap filling (Sfeir and Symington 2015; Mateos-Gomez *et al.* 2017) (Figure  2). In *C. elegans*, MMEJ is a major DSB break repair modality, and POLQ-1 (polymerase theta) has been shown to be particularly critical at regions of the genome that are hard to replicate (Koole *et al.* 2014; Roerink *et al.* 2014; van Schendel *et al.* 2016).

When extensive resection passes through internal repeat sequences, pairing of the resulting homologous single-stranded sequences can be used to heal DSBs by the SSA pathway. Following annealing, the resulting 3′-flaps are processed by the XPF/ERCC1 endonuclease. A role of *C. elegans* XPF-1 in SSA was uncovered by monitoring restoration of lacZ following induction of a DSB within two nonfunctional lacZ sequences (Pontier and Tijsterman 2009). *xpf-1* mutants enhance the phenotype of HR-defective *rad-54* mutants and also alter the repair of DSBs on the single X chromosome of males, suggesting SSA may serve a backup role in DSB repair in meiosis (Checchi *et al.* 2014; Bae *et al.* 2019).

HR is used in S and G2 phase cells, and is the predominant mode of DSB repair during meiosis. HR requires processing of the DSB, an intact DNA molecule to serve as a template to restore the missing information and resolution of the resulting JMs to generate either NCOs or COs (Figure  2).

Meiosis is unique in that excessive numbers of DNA DSBs are naturally induced by the Spo11 enzyme. HR is used to repair a minority of these breaks into COs, to shuffle maternal and paternal information, and to provide a stable link between homologous chromosomes for accurate chromosome segregation. HR intermediates that link maternal and paternal chromosomes mature into stable physical connections referred to as chiasmata. Chiasmata hold homologous chromosomes together and in conjunction with sister chromatid cohesion, withstand microtubule pulling forces for proper homolog alignment on the metaphase I spindle. Following regulated sister chromatid cohesion release, homologous chromosomes segregate away from each other at anaphase I, thereby reducing ploidy (Hillers *et al.* 2017; Láscarez-Lagunas *et al.* 2020). Consequently, successful CO formation and resolution are essential for meiosis and must be tightly regulated to ensure that the excess DSBs are repaired (as NCOs or using the sister chromatid as template) and only a subset, typically one for each chromosome pair in *C.* *elegans*, are resolved as interhomolog (IH) COs. In the subsections below, we discuss the function of *C. elegans* proteins required for HR, with a focus on meiotic DSB repair.

### Meiotic DSB formation

Meiotic recombination is initiated by the deliberate induction of DSBs by the conserved topoisomerase II-like enzyme Spo11 (Bergerat *et al.* 1997; Keeney *et al.* 1997; Dernburg *et al.* 1998) (Figure  2). The importance of DSB induction is highlighted by the ability of IR-induced DSBs to bypass the need for SPO-11 in *C. elegans* (Dernburg *et al.* 1998). During DSB formation, SPO11 becomes covalently attached to the 5′ end of the broken DNA molecule *via* a tyrosine residue (Bergerat *et al.* 1997; Keeney *et al.* 1997; Pan *et al.* 2011; Lange *et al.* 2016; Choi *et al.* 2018). The phenotypes of *C. elegans spo-11* mutants are consistent with SPO-11 being essential for meiotic DSB formation: *spo-11* mutants develop normally but lay largely dead progeny (Emb), resulting from aneuploidy. Consistent with this, approximately half of the rare survivors are males (Him) due to X chromosome nondisjunction (Dernburg *et al.* 1998). In addition, foci indicative of HR progression such as those formed by RAD-51 (Colaiácovo *et al.* 2003) and COSA-1 (Yokoo *et al.* 2012) are largely absent in *spo-11* mutants. However, occasional COSA-1 foci are detected in *spo-11* mutants in pachytene nuclei suggesting that spontaneous SPO-11-independent DNA lesions capable of recruiting meiotic DNA repair proteins occur at a low frequency in mutants lacking meiotic DSBs (Nadarajan *et al.* 2016; Machovina *et al.* 2016; Pattabiraman *et al.* 2017; Cahoon *et al.* 2019). Finally, as homologous chromosomes fail to form chiasmata, 12 univalents (as opposed to six bivalents in wild type) are observed in *spo-11* mutants at the diakinesis stage of meiosis I (Dernburg *et al.* 1998) (Figure  5B).

Unlike SPO11, DSB accessory proteins are poorly conserved. While proteins related to topoVIB, which partner with SPO11, have been identified in a number of organisms (Vrielynck *et al.* 2016; Robert *et al.* 2016), no topoV1B-like protein has been identified in *C. elegans*. Biochemical analyses of soluble *C. elegans* SPO-11 expressed in *E. coli* revealed it behaves as a monomer, but no topoisomerase activity was detected, suggesting that additional proteins (and/or post-translational modifications) may be required for catalytic activity (Yeh *et al.* 2017).

The paralogs, DSB-1 and DSB-2, which are distantly related to REC114 and DSB-3, which is distantly related to MEI4, are all required for DSB formation (Rosu *et al.* 2013; Stamper *et al.* 2013; Tessé *et al.* 2017; Hinman *et al.* 2021). *dsb-1* and *dsb-3* mutants are phenotypically similar to *spo-11* mutants, while *dsb-2* mutants have a less severe phenotype. All three proteins are associated with chromatin in a mutually dependent manner in germ cells where meiotic DSBs are induced. DSB-1, DSB-2, and DSB-3 interact with each other and in contrast to what is observed in yeast, DSB-1 interacts with SPO-11. Thus, there are both similarities and differences between SPO11 complexes and their interactions in different organisms (Rosu *et al.* 2013; Stamper *et al.* 2013; Tessé *et al.* 2017; Hinman *et al.* 2021).

Both RAD-50, an ATPase carrying a structural maintenance of chromosomes (SMC) fold, and MRE-11, a nuclease shown to have endonuclease and 3′ —> 5′ exonuclease activities in yeasts and mammalian systems (Borde 2007), are also required for DSB formation. Like *spo-11* mutants, *rad-50*, and *mre-11* mutants are Emb, Him and have 12 univalents at diakinesis (Chin and Villeneuve 2001; Hayashi *et al.* 2007). However, unlike *spo-11*, these mutants are not rescued by IR due to additional roles of RAD-50 and MRE-11 in DNA resection (see below). The specific function of RAD-50 and MRE-11 in DSB formation is not known, although the complex is likely to link distinct DNA topologies for DSB formation (Borde 2007; Kinoshita *et al.* 2009).

A host of other proteins that influence break formation, including the paralogs HIM-5 and REC-1, HIM-17, a THAP domain-containing protein implicated in chromatin complexes, XND-1, an AT-hook DNA-binding motif protein, and CRA-1, a NatB domain-containing protein that regulates acetyl levels, are chromatin associated, but in contrast to DSB-1, DSB-2, and DSB-3, chromatin association is not restricted to cells generating SPO-11-dependent DSBs (Reddy and Villeneuve 2004; Wagner *et al.* 2010; Meneely *et al.* 2012; Chung *et al.* 2015; Gao *et al.* 2015). Mutations in HIM-5, REC-1, HIM-17, XND-1, and CRA-1 lead to pleiotropic phenotypes and their role in DSB formation is likely indirect, possibly through global alteration of chromatin. Consistent with the chromatin environment influencing DSB formation and ultimately CO patterning, the spatial position of DSBs, as marked by RAD-51, is altered in *him-17* mutants such that decreased levels of RAD-51 foci on the arms and a concomitant increase of RAD-51 foci in the central region of chromosomes are observed (Nadarajan *et al.* 2021). This pattern is important as CO positioning promotes accurate chromosome segregation (Altendorfer *et al.* 2020).

In all systems examined, including *C. elegans*, the number of meiotic DSBs exceeds the number of COs. The absolute number of DSBs induced during *C. elegans* meiosis has been difficult to determine as there are no direct markers of DSBs. Estimates have varied widely, presumably due to different assays as well as different antibodies using the same assay (Mets and Meyer 2009; Hayashi *et al.* 2010; Saito *et al.* 2012); however, recent work based on high resolution cytology and multiple markers estimate that there are 4–7 DSBs/chromosome pair (Woglar and Villeneuve 2018). This number of DSBs is likely necessary to ensure that one break is repaired as an IH-CO, essential for meiotic chromosome segregation. Consequently, the majority of meiotic DSBs are resolved as NCOs or through intersister (IS) repair.

### DNA end resection

Following DSB formation by SPO-11, or as a consequence of DNA damaging agents, DSBs are either directly religated by NHEJ or processed for repair by HR (Figure  2). The decision to repair by NHEJ or HR is likely influenced by the chromatin environment; Mi2 homologs chromodomain helicase DNA-binding protein (CHD-3) and its paralog LET-418 are important for ensuring repair by HR in the germ line (Turcotte *et al.* 2018). DNA molecules not repaired by NHEJ are processed by end resection, which exposes a 3′ single-stranded tail essential for subsequent steps in recombination. Worms defective for DNA resection fail to form chiasmata, leading to high levels of Emb. Resection is mediated by the MRN complex, COM-1/CtIP (Penkner *et al.* 2007; Lemmens *et al.* 2013), the exonuclease EXO-1 (Lemmens *et al.* 2013; Yin and Smolikove 2013; Girard *et al.* 2018), and perhaps the helicase/nuclease WRN-1-DNA-2 complex (Ryu and Koo 2017) and other nucleases.

The current model, based on genetic and cytological analyses in worms as well as biochemical analyses from other systems, posits that the MRN complex binds to the broken DNA molecule and cleavage by the endonuclease activity of MRE-11 leads to the release of a short single-stranded oligonucleotide, which in the case of meiotic DSBs contains SPO-11 bound to the end (Anand *et al.* 2016). The 5′ end of the cleaved DNA strand is protected by COM-1 to prevent the NHEJ CKU-70-CKU-80 complex from binding, or if it is bound, to remove it, and thereby promote repair by HR. Consistent with this model, in the *com-1* mutant meiotic DSBs are inappropriately repaired by NHEJ, as visualized by absence of RAD-51 foci and chromatin aggregates at diakinesis. Removal of CKU-70-CKU-80, LIG-4, or the NHEJ accessory factor, NHJ-1, blocks chromatin aggregation in the *com-1* mutant (Lemmens *et al.* 2013; Vujin *et al.* 2020). However, only mutation of *cku-70* or *cku-80* restores CO formation, suggesting that COM-1 counteracts CKU-70-CKU-80 binding to promote repair by HR (Lemmens *et al.* 2013).

As MRE-11 and RAD-50 are required for both meiotic DSB formation and resection, the identification of a separation-of-function allele of *mre-11, mre-11(iow1)*, which is defective for resection but not break formation, and NBS-1, which is not required for DSB formation, have been instrumental in uncovering the role of the MRN complex in resection (Yin and Smolikove 2013; Girard *et al.* 2018). As with *com-1*, *mre-11(iow1)*, and *nbs-1* mutants inappropriately use NHEJ to repair DSBs, as visualized by chromatin aggregates at diakinesis. However, there are phenotypic differences between the *com-1*, *mre-11(iow1)*, and *nbs-1* mutants consistent with MRE-11 providing endonuclease activity and perhaps stimulating EXO-1 activity, and NBS-1 also functioning at an additional later step of recombination. Yeast two-hybrid analyses revealed that in addition to a direct interaction between MRE-11 and RAD-50, NBS-1 interacts with both MRE-11 and COM-1, thus linking these proteins in their role in resection (Girard *et al.* 2018).

There currently remains uncertainty about the contributions of other exonucleases to resection. EXO1 has been shown to be required for long range resection in yeast but not in mice (Zakharyevich *et al.* 2010; Garcia *et al.* 2011; Yamada *et al.* 2020). In *C. elegans, exo-1* single mutants have no obvious resection defects. However, in *com-1; cku-80*, *mre-11(iow1); cku-80*, and *nbs-1; cku-80* double mutants, EXO-1 becomes essential for resection, although it appears to function at different meiotic prophase stages in the different mutant combinations (Lemmens *et al.* 2013; Yin and Smolikove 2013; Girard *et al.* 2018). The contribution of the WRN-1-DNA-2 complex is even less clear. Meiotic DSBs are processed normally in the absence of this complex; however, WRN-1-DNA-2 is required for resection of IR-induced breaks (Ryu and Koo 2017). This lack of a definitive role for exonucleases in long range resection of meiotic DSBs is most likely a consequence of redundancy, and some combination of EXO-1, WRN-1-DNA-2 and perhaps other nucleases mediate long-range resection following initial MRN cleavage.

High resolution cytological analysis in *C. elegans* germ cells provides evidence for resection occurring on both sides of meiotic DSBs (Woglar and Villeneuve 2018), consistent with analysis in yeast meiosis (Brown *et al.* 2015). Bilateral resection has implications for further processing of the break.

### RAD-51-mediated strand invasion and disassembly

Central to HR is the ability of the broken DNA molecule to find and invade the nonsister or sister chromatid, leading to the displacement of the noncomplementary strand of the template duplex to form a displacement loop (D loop) (Figure  2). These events are driven by RecA recombinases and a host of mediator proteins. The extended 3′ ssDNA generated by resection is immediately coated with the single strand protein binding complex, replication protein A (RPA). The *C. elegans* genome encodes a single ortholog of RPA1 (RPA-1) and two paralogs of RPA2 (RPA-2 and RPA-4), but has no apparent RPA3 ortholog. Recent analysis suggests that RPA-1 and RPA-2 are the main components of the RPA complex that function in HR (Hefel *et al.* 2020). RPA-1 forms cytological foci in early pachytene that persists longer than RAD-51 foci, suggesting that RPA also plays a role post-strand invasion (Woglar and Villeneuve 2018).

Critical for strand invasion is the formation of the RAD-51 nucleofilament. RAD-50 has been shown to be important for RAD-51 loading during meiotic prophase, but not in proliferating germ cells (Hayashi *et al.* 2007). On the other hand, BRC-2, the ortholog of the tumor suppressor BRCA2, in combination with the conserved DSS-1 regulatory protein, plays an essential role in RAD-51 filament assembly in both meiosis and in response to DNA damage. *In vivo*, *brc-2* mutants accumulate RPA-1 on both IR-induced and meiotic DSBs and fail to load RAD-51, and BRC-2 interacts directly with RAD-51 both *in vivo* and *in vitro* (Martin *et al.* 2005). Furthermore, *brc-2* mutants are Emb and form chromatin aggregates at diakinesis that can be partially suppressed by depletion of *lig-4*, suggesting that some DSBs are repaired by NHEJ in the absence of BRC-2 (Martin *et al.* 2005; Ko *et al.* 2008). *In vitro*, bulk analyses of human BRCA2 and *C. elegans* BRC-2 indicate that BRCA2/BRC-2 can mediate strand exchange and stimulate D-loop formation (Petalcorin *et al.* 2006; Jensen *et al.* 2010; Liu *et al.* 2010). Recent work using single molecule analysis has extended this to show that BRC-2 acts primarily as an RAD-51 nucleation factor on RPA-coated ssDNA (Belan *et al.* 2021). Finally, both *in vivo* and *in vitro* analyses indicate that *C. elegans* BRC-2, but not human BRCA2, functions in SSA as worms lack an RAD52 ortholog that mediates SSA in yeast and mammals (Martin *et al.* 2005).

In most organisms, RAD51 mediates strand invasion in somatic cells, whereas both RAD51 and the meiosis-specific DMC1 recombinases are required for HR in meiotic cells. DMC1 is the major meiotic recombinase that is responsible for promoting strand invasion, while RAD51 serves an accessory role (Cloud *et al.* 2012). In *Caenorhabditis* and other closely related lineages, DMC-1 has been lost, leaving RAD-51 as the sole recombinase (Villeneuve and Hillers 2001). Interestingly, *C. elegans* RAD-51 contains DMC-1-like residues that biochemical analyses have shown promote stabilization of heteroduplex DNA, joint molecules (JMs) with mismatch containing bases, a situation likely to be encountered during meiotic recombination (Steinfeld *et al.* 2019). Furthermore, *rad-51* mutants have all the phenotypic hallmarks of being the major recombinase in meiosis: complete Emb and the appearance of chromatin aggregates at diakinesis (Rinaldo *et al.* 2002). Interestingly, three isoforms of RAD-51 are transcribed from the locus; the shorter B and C isoforms appear to be critical for recombinase function (Rinaldo *et al.* 1998; Germoglio and Adamo 2018). Unlike *brc-2*, chromatin aggregates in *rad-51* are not suppressed by *lig-4* mutation, suggesting that an alternative pathway other than NHEJ promotes chromosomal fusions in the *rad-51* mutant, and this alternative pathway is dependent on BRC-2, perhaps in its role in SSA (Martin *et al.* 2005).

A Shu complex composed of RAD-51 paralogs RFS-1 and RIP-1, and the SWIM domain containing protein SWS-1, likely functions as an RAD-51 mediator complex. *rfs-1*, *rip-1*, and *sws-1* are sensitive to DNA damaging agents and have mild Emb and Him phenotypes, consistent with a supporting role in meiotic recombination, and the corresponding proteins form a complex (Ward *et al.* 2007; Yanowitz 2008; Taylor *et al.* 2015; McClendon *et al.* 2016). Biochemical analyses suggest that the Shu complex remodels the RAD-51 filament to promote strand invasion and D loop formation (Taylor *et al.* 2015). Recent single molecule analyses revealed that RFS-1 and RIP-1 promote RAD-51 3′–5′ filament growth (Belan *et al.* 2021). A mediator function of the RAD54 helicase has been suggested based on RAD54-mediated D-loop formation *in vitro* (Wright and Heyer 2014). *rad-54* mutants are complete Emb, accumulate RAD-51 foci, and have chromatin aggregates at diakinesis (Mets and Meyer 2009; Ward *et al.* 2010).

There is clear evidence that the nature of the DSB affects RAD-51 nucleofilament formation. The RAD-51 paralog RFS-1 is required for RAD-51 focus formation upon treatment with DNA crosslinking agents (cisplatin and nitrogen mustard) as well as camptothecin, a topoisomerase I poison that inhibits the enzyme and prevents its release from DNA, thus creating capped single-ended DSBs (Ward *et al.* 2007). In contrast, RFS-1 is not required for RAD-51 focus formation upon IR, which generates canonical DSBs. In agreement with this, *rfs-1* mutants are exquisitely sensitive to DNA crosslinking agents and camptothecin, while only moderately sensitive to IR (Ward *et al.* 2007). Consistent with current models of ICL repair, where MUS-81 and XPF-1 nucleases are required for the unhooking of crosslinked DNA to generate single-ended DSBs funneled into HR, these nucleases are also required for RAD-51 focus formation (Ward *et al.* 2007; Deans and West 2011; Ceccaldi *et al.* 2016; Hashimoto *et al.* 2016). Interestingly, DNA replication blockage by crosslinking agents and camptothecin is distinct from the DSBs generated by replication fork stalling mediated by HU, which triggers the DNA replication checkpoint and requires ATR (Ward *et al.* 2007) (see below, *The DNA replication checkpoint*).

Following strand invasion and D-loop formation, RAD-51 filaments must be removed from the dsDNA. Many of the same mediators that promote RAD-51 remodeling and D-loop formation also facilitate the disassembly of the RAD-51 filament. The weak Emb and Him phenotypes of the *rsf-1*, *rip-1*, and *sws-1* Shu mutants are enhanced by mutation of the helicase, HELQ-1 (Ward *et al.* 2010; McClendon *et al.* 2016). Double mutants of Shu and *helq-1* lead to high levels of Emb, chromosome aggregates at diakinesis and retention of RAD-51 through late meiotic prophase. Biochemical analyses indicate that RSF-1 and HELQ-1 disassemble RAD-51 from dsDNA using distinct mechanisms (Ward *et al.* 2010). RAD54 orthologs have also been shown to promote the disassembly of the RAD51 filament *in vitro* (Wright and Heyer 2014). Thus, RAD-54 is likely to promote both D loop formation and disassembly of the RAD-51 filament in *C. elegans*.

*In vitro*, Bloom RECQ-like helicase (BLM/HIM-6) can disassemble D loops (van Brabant *et al.* 2000; Bachrati *et al.* 2006) and another RECQ-like helicase (RCQ-5) can disrupt the RAD51 filament (Hu *et al.* 2007). *him-6* mutants are radiation sensitive and have ∼50% Emb, Him, and a mixture of bivalents and univalents in diakinesis nuclei, suggesting both positive and negative roles in CO formation (Zetka and Rose 1995; Wicky *et al.* 2004; Schvarzstein *et al.* 2014). HIM-6 protein is present early in meiotic prophase at multiple foci and as prophase progresses, the protein concentrates at IH recombination sites, consistent with HIM-6 functioning at multiple steps of DSB repair (Woglar and Villeneuve 2018). Single molecule analyses of recombinant HIM-6 provide evidence for a reiterative mode of DNA unwinding and rewinding. In the presence of RPA, DNA unwinding is unidirectional and processive (Choi *et al.* 2019). Cytologically, RPA is present following RAD-51 disassembly, and is observed adjacent to HIM-6 foci (Woglar and Villeneuve 2018), suggesting that RPA may influence the unwinding activity of HIM-6 *in vivo*. In contrast to *him-6*, *rcq-5* mutants display no obvious meiotic phenotype but are sensitive to IR (Jeong *et al.* 2003). However, removal of RTEL-1, another helicase that mediates disassembly of preformed D loop in the absence of RAD-51 *in vitro*, in *him-6* or *rcq-5* mutants leads to high levels of RAD-51 throughout the germ line and an enhanced Emb phenotype, consistent with a role in dismantling D loops (Ward *et al.* 2010). RTEL-1 is proposed to mediate synthesis-dependent strand annealing (SDSA), whereby one DSB end invades a homolog and primes DNA synthesis. The nascent strand is then displaced and anneals to complementary sequences on the second DSB end, leading to the formation of NCOs (Figure  2). Consistent with this, in the absence of RTEL-1, more strand invasions are processed into COs, leading to an expanded genetic map (Youds *et al.* 2010).

### Pro-CO factors and resolution of JMs

Following strand invasion and removal of RAD-51, the invading strand primes DNA synthesis using the intact nonsister (or sister) chromatid as a template to restore genetic material lost by resection. Subsequently, the resected second-end pairs with the displaced strand (second-end capture) and is also extended by DNA synthesis. Second-end capture leads to formation of a double Holliday junction (HJ), which can be resolved to generate CO or NCO products (Figure  2).

A number of meiosis-specific CO promoting proteins have been identified and include the conserved MutSγ complex composed of HIM-14/MSH-4 and MSH-5, the cyclin-related protein COSA-1, and the E3 ligase heterodimer ZHP-3-ZHP-4. *him-14, msh-5, cosa-1, zhp-3*, and *zhp-4* mutants are defective for CO formation and fail to form chiasmata, as evidenced by 12 univalents at the diakinesis stage, resulting in Him and Emb phenotypes (Zalevsky *et al.* 1999; Kelly *et al.* 2000; Jantsch *et al.* 2004; Bhalla *et al.* 2008; Yokoo *et al.* 2012; Zhang *et al.* 2018; Nguyen *et al.* 2018). The corresponding proteins ultimately all congregate in a mutually dependent manner at six foci, one per homolog pair at late pachytene, marking the single CO that will become the chiasma. High resolution microscopy allowed for further resolution of these foci into distinct subcomplexes, such that MSH-5 doublets are positioned orthogonally to HIM-6 and RMH-1/RMI1 doublets (Jagut *et al.* 2016; Woglar and Villeneuve 2018). This configuration of MSH-5 is consistent with a model supported by *in vitro* work that posits that MutSγ binds JM structures in tandem and stabilizes HJs in a conformation that is refractory to branch migration, which would lead to dissolution and NCOs (Snowden *et al.* 2004; Lahiri *et al.* 2018). COSA-1, which contains a diverged cyclin fold, likely acts with a cell cycle related kinase to phosphorylate proteins, while ZHP-3-ZHP-4 is a predicted SUMO or ubiquitin E3 ligase that modifies proteins, suggesting that COSA-1 and ZHP-3-ZHP-4 serve regulatory roles in CO designation.

Another E3 ligase heterodimer, ZHP-1-ZHP-2, both restricts COs and promotes CO maturation (Zhang *et al.* 2018). *zhp-1* and *zhp-2* mutants show elevated levels of RAD-51 and pro-CO factors in meiotic prophase, with a mix of bivalents and univalents at diakinesis. This dual negative and positive role in CO regulation is shared by HIM-6, RMH-1, and RMIF-2/RMI2, members of the RecQ helicase–topoisomeraseIII–Rmi1 (RTR) complex along with TOP-3 (Wicky *et al.* 2004; Schvarzstein *et al.* 2014; Jagut *et al.* 2016). HIM-6, RMH-1, and RMIF-2 also concentrate at CO designation sites, and reduced numbers of COs are observed in the corresponding mutants, consistent with a role in CO formation. However, *in vitro* the RTR complex can dismantle double HJs leading to NCOs: BLM helicase can mediate branch migration to bring the two HJs in close proximity, allowing topoisomerase 3 stimulated by the scaffolding proteins RMI1/2 (RMH-1/RMIF-2) to unhook the two DNA strands by decatenation (Bizard and Hickson 2014). Thus, HIM-6, RMH-1, and RMIF-2 serve both pro- and anti-CO roles.

Resolution of HJs to form COs is mediated by the redundant activities of structure-specific endonucleases, in partnership with other proteins. One activity consists of the XPF-1 nuclease in combination with the HIM-6 helicase (Agostinho *et al.* 2013; O’Neil *et al.* 2013; Saito *et al.* 2013). It has been proposed that unwinding of an HJ by HIM-6 could generate a substrate for XPF-1 cleavage. The other activity is composed of the nucleases MUS-81, presumably in complex with EME-1, and SLX-1 (Agostinho *et al.* 2013; O’Neil *et al.* 2013; Saito *et al.* 2013); sequential action of SLX-1 to generate a nicked HJ, which in turn is the preferred substrate for MUS-81, leads to HJ resolution *in vitro* (Wyatt *et al.* 2013). Both of these complexes function with the SLX-4/HIM-18 scaffold, which likely coordinates the different biochemical activities. Single mutants in any of these genes (except for *slx-4*) are sensitive to DNA damaging agents but have only mild meiotic phenotypes. However, when double mutants for each redundant activity are generated, there are significant Emb and Him phenotypes and a reduction in CO levels. Pro-CO factors still concentrate at six foci in the double mutants, consistent with a role postdesignation and specifically in CO resolution. The restructuring of the bivalent around the CO site still occurs in double mutants defective for HJ resolution, suggesting that CO initiation, but not resolution, is likely required for this process (Agostinho *et al.* 2013). Further, abnormal bivalents are observed at diakinesis consistent with a defect in resolution (Agostinho *et al.* 2013; O’Neil *et al.* 2013; Saito *et al.* 2013). Interestingly, these redundant activities do not account for resolution of all JMs as there are still significant levels of COs in the double mutants. While the LEM-3/ANKLE-1 nuclease was identified as being synthetically lethal with MUS-81 and SLX-1 (but not XPF-1), it does not appear to be required for CO formation *per se* (Hong *et al.* 2018b), suggesting that there are still resolution activities awaiting identification.

While the focus of meiotic recombination is centered on the formation of IH-COs for chiasma formation and accurate chromosome segregation, all DSBs must be repaired. Given that ∼4× the number of DSBs are induced than will become COs, many meiotic breaks are repaired as NCOs. As illustrated in Figure  2 and discussed above, there are multiple branches of the recombination pathway that mediate NCO outcomes, such as SDSA and HJ dissolution. Additionally, meiotic recombination intermediates can be repaired *via* the sister chromatid, the predominant template used in somatic cells. The structural maintenance complex SMC-5-SMC-6, and the E3 ubiquitin ligase BRC-1-BRD-1 are important for repair of intermediates through the IS pathway. Mutants in both of these complexes are sensitive to DNA damaging agents and have mild Emb and Him phenotypes, and some RAD-51 foci perdure until late pachytene, suggesting a subset of recombination intermediates are not efficiently processed (Boulton *et al.* 2004; Bickel *et al.* 2010; Wolters *et al.* 2014). In *brc-1* and *brd-1* mutants, COSA-1 concentrates at six foci as in wild-type, and six bivalents are observed at diakinesis (Adamo *et al.* 2008; Bickel *et al.* 2010; Janisiw *et al.* 2018; Li *et al.* 2018). However, under conditions where IH recombination is blocked, removal of these complexes results in chromosome fragments at diakinesis, consistent with a block in DSB repair *via* the sister (Adamo *et al.* 2008; Bickel *et al.* 2010). Thus, the IS pathway is important for repair of those DSBs not channeled through IH COs or NCOs. Recent work using differential labeling of sister chromatids and a reporter system for intrachromatid and IS recombination have revealed that the majority of IS events are resolved as NCOs (Almanzar *et al.* 2020; Toraason *et al.* 2020). Finally, as cells progress to the end of prophase, any remaining breaks must be repaired prior to the meiotic divisions. NHEJ, SSA, and MMEJ have all been shown to be engaged at the end of meiotic prophase when meiosis is compromised (Smolikov *et al.* 2007; Macaisne *et al.* 2018).

Analyses in *C. elegans* have also revealed the importance of chromosome structure in repairing DSBs by HR. In the absence of SMC-5-SMC-6, BRC-1-BRD-1 promotes toxic recombination intermediates in response to replication fork stalling (Wolters *et al.* 2014). In addition, BRC-1-BRD-1 promotes aberrant recombination intermediates as visualized by chromatin bridges in the absence of both SMC-5-SMC-6 and HIM-6. This is likely due to defects in chromatin structure as *brc-1* or *brd-1* mutants also suppress meiotic chromatin bridges observed when HCP-6, a component of condensin II, is depleted (Hong *et al.* 2016). Thus, successful repair by HR requires both the enzymatic machinery and proper chromosome structure.

### DSB repair in the male germ line

DSB repair has been primarily studied in oogenic hermaphrodite germ lines due to the attributes discussed above. However, a few studies have examined DSB repair in males and have provided insight into how sex influences the induction and processing of DSBs. While the same machinery appears to be required for the different steps of DSB formation and processing, there are differences in the regulation of these events. The pattern of meiotic RAD-51 foci is distinct in the male *vs* hermaphrodite germ line: RAD-51 appears to load earlier in meiotic prophase, reaches higher steady state levels, and is removed more abruptly (Jaramillo-Lambert and Engebrecht 2010; Checchi *et al.* 2014). This pattern suggests that more DSBs are induced and that repair occurs with faster kinetics in male compared to female germ cells. Interestingly, *brc-1* and *brd-1* mutants display different RAD-51 phenotypes in spermatogenesis *vs* oogenesis. In male meiosis, fewer RAD-51 foci are observed in early meiotic prophase, while RAD-51 foci perdure in late meiotic prophase in the hermaphrodite germ line (Adamo *et al.* 2008; Janisiw *et al.* 2018; Li *et al.* 2018, 2020). The reduction in RAD-51 foci in the absence of BRC-1-BRD-1 in males can be suppressed by mutation of NHEJ proteins, suggesting that BRC-1-BRD-1 promotes repair by HR perhaps through regulating DNA end resection (Li *et al.* 2020). In addition to sex-specific regulation of meiotic DSB processing, topoisomerase 2/TOP-2 has an unique role during spermatogenic chromosome segregation; TOP-2 is most likely required to deal with the tight compaction of the sperm DNA (Jaramillo-Lambert *et al.* 2016; Bhandari *et al.* 2020). Future work analyzing male meiosis will provide insight into how sex influences the induction and processing of DSBs.

Spermatogenesis, but not oogenesis, has recently been shown to be sensitive to small increases in temperature, leading to formation of SPO-11-independent DSBs. These DSBs can be processed into COs, as marked by COSA-1. Heat-induced DSBs appear to be a consequence of mobility of Tc1/mariner transposable elements (Kurhanewicz *et al.* 2020). Why heat stress induces transposon activity specifically in spermatogenesis is not known but may be a consequence of differences in chromatin state, and/or sex-specific gene expression patterns. On the other hand, piRNAs are needed for repressing transposon activity and the associated DNA damage during oogenesis, as mutants defective for piRNA synthesis show excessive apoptosis induction in hermaphrodites (Manage *et al.* 2020).

## DDR, checkpoint signaling, fail-safe mechanisms, and apoptosis induction

DNA repair and recombination are intimately linked to checkpoint signaling pathways, which monitor and delay or arrest the cell cycle to coordinate repair with division, or induce apoptosis. A number of different checkpoint pathways that use both common and specific sensors and effectors recognize different lesions at different stages of the cell cycle. *C.* *elegans* contains all of the major signaling pathways and has been instrumental in elucidating the intricacies of DNA damage checkpoints in the germ line, as well as other fail-safe mechanisms important for maintaining genome integrity.

### The DNA replication checkpoint

The DNA replication checkpoint operates in S-phase and is activated when obstacles in DNA stall replication forks (Labib and De Piccoli 2011; Técher *et al.* 2017). Cell cycle arrest/delay is mediated by the conserved ATR checkpoint pathway, upstream sensors, such as RPA-1, WRN-1 helicase, HPR-17/9–1–1 clamp loading complex, and the CHK-1 mediator kinase (Stevens *et al.* 2016) (see *Germ cell DNA damage checkpoint pathways*). Interestingly, cells of different fates respond differently to DNA replication stress (Brauchle *et al.* 2003). The posterior daughter of the zygote, namely the P1 germ cell, is particularly sensitive to the replication checkpoint. Indeed, the replication checkpoint delays DNA replication even under unperturbed conditions, which contributes to the differential timing of AB and P1 cell divisions. The pace of the first three rounds of embryonic cell divisions is increased upon *atl-1/chk-1* RNAi depletion (Moser *et al.* 2009), in line with the replication checkpoint being used to regulate developmental timing. A recent study revealed that replication stress promotes cell elimination by extrusion in the embryo and is also dependent on ATR and CHK-1 (Dwivedi *et al.* 2021).

### Fail-safe mechanisms acting during embryogenesis: NoCut checkpoint and LEM-3

To ensure faithful genome inheritance, chromatids must be properly segregated to daughter cells, which requires the removal of all physical connections between sister chromatids before cells divide. Besides cohesins, which act as a proteinaceous glue, a variety of other DNA-mediated chromatid connections have to be removed. These include intermediates of DNA recombination such as HJs, points at which chromatids have become intertwined, and loci that have not been replicated by the time cells reach the metaphase–anaphase transition. The conserved NoCut checkpoint delays cytokinesis progression when persistent DNA bridges occur to allow for their processing (Steigemann *et al.* 2009; Bembenek *et al.* 2013) [for reviews, see Amaral *et al.* (2017) and Hong *et al.* (2021)]. The *C. elegans* LEM-3 nuclease was recently shown to have a role in processing DNA intermediates at chromatin bridges right before the first zygotic cell division (Hong *et al.* 2018a). LEM-3 acts at the midbody, the structure where abscission occurs at the end of cytokinesis. LEM-3 localization depends on the assembly of the central spindle and the midbody, and also requires the AIR-2 Aurora B kinase. Interestingly, maintaining the NoCut checkpoint also requires activated Aurora B kinase (Steigemann *et al.* 2009; Bembenek *et al.* 2013; Amaral *et al.* 2017; Hong *et al.* 2021). This, and the finding that cytokinesis progresses faster in *lem-3* mutants suggests that LEM-3 is part of the NoCut pathway. It remains to be seen if the mammalian ortholog Ankle1 has a similar role as LEM-3 (Braun *et al.* 2016), but consistent with LEM-3 processing a large variety of DNA intermediates, Ankle1 cleaves multiple branched DNA structures *in vitro*, including HJs (Song *et al.* 2020). LEM-3 acts cooperatively with BRC-1-BRD-1 to promote genome integrity (Hong *et al.* 2018a), which might provide a molecular basis for the suspected role of ANKLE1 in human breast cancer (Lawrenson *et al.* 2016; Tian *et al.* 2020).

### Germ cell DNA damage checkpoint pathways

Most studies on checkpoint signaling are focused on the germ line. Within the gonad, DNA damage checkpoint responses are spatially separated; DNA damage-induced cell cycle arrest or delay occurs solely in mitotically proliferating cells, while checkpoint-induced apoptosis only affects late stage pachytene germ cells (Gartner *et al.* 2000). DNA damage checkpoints are triggered by a variety of DNA damaging agents, which ultimately cause excessive ssDNA and/or DSBs. Given that large numbers of DSBs are induced during meiosis, and that DSBs exceed the number of CO events, the DNA damage checkpoint is also used to monitor the repair and resolution of SPO-11-induced DSBs (Gartner *et al.* 2000). In late stage pachytene cells, where apoptosis occurs, the designation of one DSB as a CO is largely completed and any remaining DSBs have to be repaired. This occurs *via* HR using the sister chromatid as a repair template, while end-joining mechanisms serve a backup role (see *DSB repair*). Defects in processing of DSBs result in elevated apoptosis induced by the pachytene checkpoint (Gartner *et al.* 2000). Repair of SPO-11-induced breaks requires the pairing and synapsis of homologous chromosomes (Alpi *et al.* 2003; Colaiácovo *et al.* 2003). Pachytene checkpoint-induced apoptosis is, therefore, also induced when meiotic chromosomes fail to synapse. A second, genetically separable apoptosis-inducing pathway, the synapsis checkpoint, is specifically needed to monitor meiotic chromosome pairing and synapsis and requires the AAA-ATPase PCH-2 (Bhalla and Dernburg 2005; Deshong *et al.* 2014). The histone methyltransferase complex, DOT-1.1-ZFP-1, which controls H3K79me levels in the germ line, functions in the synapsis checkpoint independently of PCH-2, suggesting that the synapsis checkpoint is controlled in part by the chromatin landscape (Láscarez-Lagunas *et al.* 2020). Finally, when the early stages of meiotic recombination and chromosome pairing, which normally occur in the transition zone, are compromised, the exit from the transition zone is delayed (Stamper *et al.* 2013; Woglar *et al.* 2013; Silva *et al.* 2014). This latter pathway requires CHK-2, a kinase involved in mediating DNA damage signaling in response to DSBs in most organisms, but co-opted as a master regulator of the initiation of meiotic recombination and chromosome pairing in *C. elegans* (Kim *et al.* 2015) [for review, see Hillers *et al.* (2017)].

The DNA damage checkpoint pathway leading to germ cell cycle arrest/delay or apoptosis requires the same upstream components as in mammalian cells. In *C. elegans*, DDRs are largely mediated by ATL-1 (Aoki *et al.* 2000; Garcia-Muse and Boulton 2005), the worm ATR homolog. However, despite ATR functioning with the adapter ATRIP in most systems, no ATRIP-like molecule has been described in *C. elegans*. The related ATM-1 kinase has only a minor role in DNA damage signaling, but like its yeast counterpart, also functions in telomere maintenance (Jones *et al.* 2012). Interestingly, ATM-1 seems to have a role in triggering cell death of postmitotic intestinal cells, possibly associated with autophagy (Moriwaki *et al.* 2018).

A number of proteins work with ATR/ATM to facilitate checkpoint signaling. CLK-2/TEL2 was first implicated as a checkpoint protein in *C. elegans* (Ahmed *et al.* 2001). Later studies using mammalian cells showed that CLK-2/TEL2 acts as a chaperonin for PI3 kinases, including ATM and ATR (Vaughan 2014; Sugimoto 2018). Upstream checkpoint signaling also involves the conserved DNA damage-specific clamp loader, RAD17, which recruits a proliferating cell nuclear antigen-like RAD9-RAD1-HUS1 complex, referred to as “9–1–1,” to the dsDNA–ssDNA junction at resected DNA ends. The same complex is also required for telomere maintenance (Ahmed and Hodgkin 2000; Gartner *et al.* 2000; Boulton *et al.* 2002). *C.* *elegans* ZTF-8, the homolog of human RHINO, partially colocalizes with HUS-1 foci in response to IR, and some HUS-1 and ZTF-8 localization is interdependent (Kim and Colaiácovo 2014). In addition, ZTF-8 interacts with MRT-2, the *C. elegans* ortholog of *S. pombe* Rad1, by yeast two-hybrid assays. ZTF-8 is required for full IR-induced pachytene cell apoptosis. In contrast, the DNA replication checkpoint in mitotic cells is activated normally in *ztf-8* mutants. ZTF-8 transiently accumulates in the nucleolus upon IR treatment, perhaps suggesting a role in repairing ribosomal DNA repeats (Kim and Colaiácovo 2014). 9–1–1 loading and checkpoint-induced apoptosis require SCC-2 and SCC-3 cohesins subunits, cohesin also being required for efficient DSB repair of IR and SPO-11 induced DSBs, as is the case in yeast and mammalian cells (Lightfoot *et al.* 2011).

Checkpoint signaling through ATR/ATM leads to the activation of the CHK-1 kinase, which in turn activates downstream effectors of the checkpoint signaling pathway (Brauchle *et al.* 2003; Jaramillo-Lambert *et al.* 2010). In mammals, the checkpoint protein 53BP1 is phosphorylated by ATM and required for checkpoint activation and DNA end-joining (Shibata and Jeggo 2020). Mutants of the *C. elegans* homolog *hsr-9* are moderately defective for DNA damage-induced apoptosis, but fully proficient for cell cycle arrest (Ryu *et al.* 2013). *hsr-9* mutant strains are not hypersensitive to IR, but suppress the hypersensitivity of lines depleted for RAD-54, suggesting a connection between HSR-9 and HR. The conserved WRN-1 and HIM-6 helicases related to human Werner’s and Bloom’s helicases, mutations of which lead to accelerated aging and cancer, have a role in checkpoint signaling in response to DNA replication stress (Wicky *et al.* 2004; Lee *et al.* 2010a). Finally, *C. elegans* mutants defective for the conserved GEN-1 HJ resolvase are also defective for checkpoint-induced cell cycle arrest and apoptosis (Bailly *et al.* 2010). Given that this enzyme is involved in processing late stage recombination intermediates, GEN-1 might serve as the link between defective recombination and checkpoint signaling.

The specialized structure of meiotic chromosomes necessitates checkpoint-dependent adaptations to ensure genome integrity upon excessive DSB formation. SPO-11-induced DSBs are important to facilitate CO formation; however, excessive DSBs formed by SPO-11 or upon IR exposure need to be repaired using the sister chromatid and not the homologous chromosome as a repair template. Two mechanisms that facilitate such checkpoint-dependent sister chromatid repair have been described (Couteau and Zetka 2011; Garcia-Muse *et al.* 2019). Synapsed chromosomes have the capability to locally desynapse when excessive DSBs occur, and this is correlated with the loss of histone H2AcK5 (Couteau and Zetka 2011). Localized desynapsis is compromised upon depletion of the MYS-1 subunit of the TIP60 histone acetyltransferase. While MYS-1 or ATM-1 deficiency does not alter the basal level of H2AcK5 or its reduction upon IR, the reacquisition of H2AcK5 after IR treatment and synapsis restoration require both MYS-1 and ATM-1 (Couteau and Zetka 2011). A more recent study provides evidence that synapsis proteins are direct targets of ATR/ATM DNA damage-induced phosphorylation. Six putative phosphorylation sites were identified in the SC protein SYP-1, and phosphorylation site mutants show heightened sensitivity to IR and increased apoptosis induction. Consistent with ATR/ATM phosphorylation being required to channel HR toward the sister chromatid, embyronic lethality is enhanced in the *syp-1* phosphorylation site mutants in the absence of BRC-1 (Garcia-Muse *et al.* 2019), which is required for IS repair (Adamo *et al.* 2008).

ATM-1 signaling as measured by damage dependent pS/TQ phosphorylation is restricted to the meiotic part of the germ line, suggesting that checkpoint signaling in the germ line is under developmental control (Vermezovic *et al.* 2015). Interestingly, the GLP-1/Notch receptor maintains the mitotic germ cell fate and appears to directly inhibit ATM. When Notch signaling is blocked, increased ATM signaling occurs, and conversely, reduced ATM signaling is observed in GLP-1 gain-of-function mutants. In mammalian cells, Notch directly binds and inhibits ATM-1, suggesting a conserved role for this interaction (Vermezovic *et al.* 2015).

Genetic screens designed to identify checkpoint components have uncovered roles for nonsense-mediated mRNA decay (NMD) in promoting efficient DSB repair. Mutants defective in various genes required for NMD are exquisitely IR sensitive and show delayed DSB repair. NMD is a conserved pathway that eliminates mRNAs that contain premature stop codons (González-Huici *et al.* 2017). The pathway is also used for fine tuning mRNA expression. Interestingly, the apical component of this pathway, the SMG-1 PI3-kinase, is highly related to ATM and ATR checkpoint kinases. Further, the CLK-2/TEL2 PI3-kinase chaperone has a role in both DNA damage checkpoint signaling and NMD, suggesting these pathways may be linked (Guo *et al.* 2021). It will be interesting to determine whether the NMD pathway directly affects DNA repair, or if perturbation of DNA repair gene expression causes heightened IR sensitivity.

The mitotic spindle assembly checkpoint (SAC) pathway allows transition into anaphase when all kinetochores are attached to the spindle. Interestingly, otherwise viable mutations in SAC genes have heightened sensitivity to IR and other DNA damaging agents (Lawrence *et al.* 2015; Bertolini *et al.* 2017). This might be caused by precocious anaphase entry in the presence of otherwise sublethal doses of IR-induced DNA damage. Alternatively, or in addition, components of the SAC might directly impinge on DNA repair. In line with this hypothesis, the *C. elegans* MAD-2 SAC protein and the histone variant CENPA/HCP-3 become enriched with RAD-51 foci at the nuclear periphery in a DDR-dependent manner (Lawrence *et al.* 2015). These results suggest that SAC functions with the DDR to facilitate repair of DNA damage.

The meiotic pachytene checkpoint is also induced when compromised mitotic germ cells progress into meiosis (Stevens *et al.* 2013). This phenomenon was observed when the spindle was perturbed in mitotic germ cells using temperature sensitive mutants affecting kinetochore formation, mitotic spindle assembly or centrosome duplication. At the restrictive temperature, these mutants activate the SAC and undergo transient cell cycle arrest. However, compromised cells eventually enter meiosis and are eliminated by the pachytene checkpoint. Checkpoint-induced apoptosis requires that compromised cells transiting into the meiotic compartment are subjected to elevated levels of SPO-11-induced breaks, possibly caused by defects in proper meiotic chromosome alignment and pairing. When apoptosis is blocked in *cep-1* (see below) and spindle-defective double mutants, aneuploidy is increased, as observed in diakinesis nuclei, consistent with the pachytene checkpoint eliminating aneuploid cells that arose in mitosis (Stevens *et al.* 2013).

In conclusion, DDR pathways mainly act in the germ line and use conserved signaling molecules to monitor both the normal progression of HR as well as to sense and respond to different types of DNA lesions.

### Checkpoint-induced apoptosis

**Figure 6:**
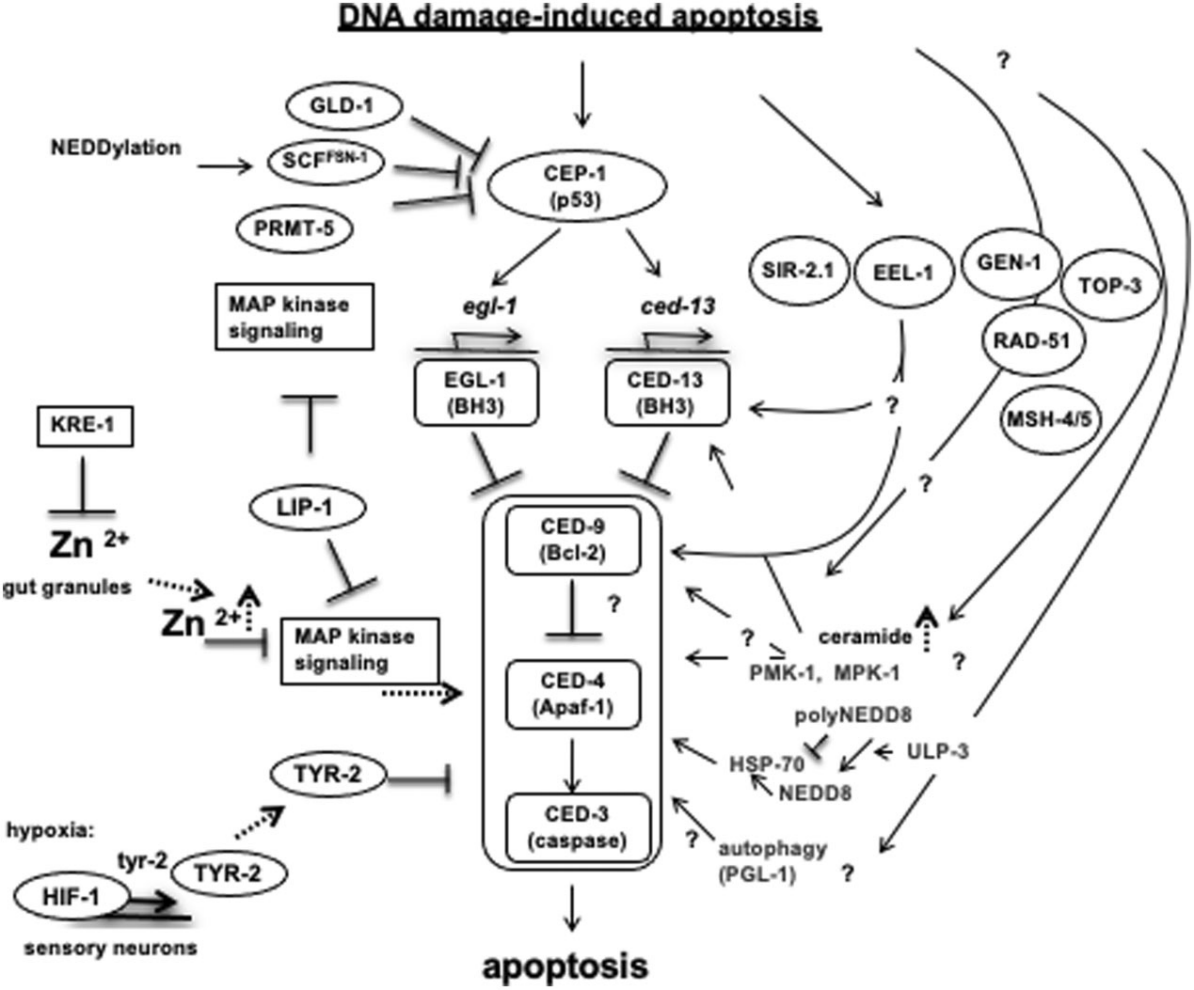
DNA damage-induced apoptosis. Proteins and pathways involved in DNA damage-induced apoptosis induction are indicated. A core, conserved CEP-1/p53 dependent germ cell apoptosis pathway is activated by the DNA damage checkpoint and leads to the transcriptional induction of *egl-1* and *ced-13* genes needed for CED-9 inactivation, and the activation of the core apoptosis pathway composed of CED-4 and CED-3. Multiple pathways as positive or negative regulators of apoptosis function in parallel to CEP-1.

*C.* *elegans* checkpoint-induced apoptosis requires the same conserved core apoptosis pathways used during development (Conradt and Xue 2005; Conradt 2009; Bailly and Gartner 2013) (Figure  6). This includes the antiapoptotic Bcl2-like protein CED-9, and the proapoptotic Apaf-1-like CED-4 protein, which acts as a scaffold for the activation of the CED-3 caspase; CED-3 ultimately triggers the demise of apoptotic cells. DNA damage-induced apoptosis requires CEP-1-dependent transcriptional induction of the redundant *egl-1* and *ced-13* encoding BH3 domain-only proteins; CEP-1 is the sole *C. elegans* p53 family member (Derry *et al.* 2001; Schumacher *et al.* 2001). EGL-1 and CED-13 act by directly binding to CED-9, where they have been proposed to displace CED-9-bound CED-4. Free CED-4 has been proposed to act as a scaffold for CED-3 caspases through an induced proximity mechanism (Chen *et al.* 2000). However, such a mechanism is unlikely to occur in *C. elegans* germ cells as CED-4 is largely located in the cytoplasm and becomes enriched at the nuclear periphery concomitant with apoptosis induction, while CED-9 is associated with the outer mitochondrial membrane (Pourkarimi *et al.* 2012).

Although mechanisms of apoptotic execution have diverged, the basic pathway of apoptosis induction appears to be conserved. CEP-1 is most closely related to the mammalian p63 p53 family member, and in female mammalian germ cells p63 is required to trigger the apoptosis of damaged germ cells through the transcriptional induction of the BH3 domain only proteins, Puma and Noxa (Suh *et al.* 2006; Rutkowski *et al.* 2010; Kerr *et al.* 2012). Several mechanisms regulate CEP-1 and EGL-1 activity in the germ line. *egl-1* translation is repressed by the *mir-35* miRNA (Doll *et al.* 2019; Tran *et al.* 2019), and by GLD-1, which binds *cep-1* mRNA and represses translation in early and midpachytene (Schumacher *et al.* 2005b). In *gld-1* mutants that fail to bind to *cep-1* mRNA, apoptosis induction occurs more distally in the germ line, from mid pachytene onwards. DNA damage-induced apoptosis is also integrated with MAP kinase signaling, which is induced upon treatment with IR and required for DNA damage-dependent apoptosis induction (Rutkowski *et al.* 2011). Conversely, *cep-1*-dependent apoptosis is increased in mutants defective for the MAP kinase phosphatase LIP-1. However, elucidating the specific requirement for MAP kinase signaling in apoptosis is complicated by the pleiotropic nature of MAP kinase signaling in late stage oocyte development (Arur *et al.* 2009, 2011; Perrin *et al.* 2013; Nadarajan *et al.* 2016; Achache *et al.* 2019). Intriguingly, the *C. elegans* MPK-1 MAP kinase appears to act in the same apoptotic induction pathway as the RPOA-2 subunit of RNA polymerase I; the apoptotic role of the essential *rpoa-2* gene was revealed by a hypomorphic mutation (Eberhard *et al.* 2013). Protein arginine methyltransferase *5* (PRMT-5) acts as a negative regulator of *cep-1*, likely by binding and methylating the conserved p53/CEP-1 transcriptional cofactor CBP-1 (Yang *et al.* 2009). Finally, the cullin3 SCF^FSN-1^ complex is required to dampen the apoptotic response, by directly or indirectly affecting CEP-1 turnover; CEP-1 protein levels are increased in *fsn-1* mutants (Gao *et al.* 2008). In summary, a conserved checkpoint pathway involving the CEP-1 transcription factor and the transcriptional induction of EGL-1 and CED-13 BH3-domain proteins are required for apoptosis induction.

Full apoptosis induction depends on autophagy (Wang *et al.* 2013), and genetic evidence suggests that the autophagic removal of P-granules, conserved germline-specific nonmembrane-bound ribonucleoprotein organelles, is required for the full induction of germ cell apoptosis upon DNA damage (Min *et al.* 2016, 2019). Several autophagy genes are transcriptionally activated following DNA damage, and this activation requires CEP-1, suggesting that autophagy is an integral part of DNA damage-induced apoptosis induction (Min *et al.* 2019). However, autophagy appears to have pleiotropic effects as DNA damage-independent germ cell apoptosis is increased in autophagy defective mutants, while DNA damage-induced apoptosis is compromised (Min *et al.* 2019). The loss of the P granule endoribonuclease PGL-1 occurs very early during *C. elegans* germ cell apoptosis under both physiological and DNA damage-induced conditions, likely as a decisive and irreversible event (Raiders *et al.* 2018; Min *et al.* 2019).

Interestingly, apoptosis induction fails to occur in male germ lines, despite *cep-1*-dependent *egl-1* and *ced-13* induction, and expression of proapoptotic core proteins CED-4 and CED-3 (Gartner *et al.* 2000; Jaramillo-Lambert *et al.* 2010). The restriction of apoptosis induction to female germ cells depends on germline sex but not sex chromosomes or somatic sex (Jaramillo-Lambert and Engebrecht 2010). Analyzing apoptosis induction in germ lines of sex-determination mutants that are XO females (Checchi and Engebrecht 2011), found that DNA damage-induced apoptosis, likely triggered by SPO-11-dependent DSBs, is hyper-induced in the absence of the histone H3K9 methyltransferase MET-2. In *met-2* mutants, the single X chromosome becomes transcriptionally active as evidenced by increased cytological staining for the transcriptional markers histone H3K4me2 and RNA Pol2 phosphoSer5. These data, corroborated by the finding that DSBs induced in heterochromatic extrachromosomal arrays fail to trigger DNA damage signaling, suggest that heterochromatin is able to block checkpoint signaling.

During somatic development, apoptosis induction is largely mediated by the cell-type-specific transcriptional regulation of *egl-1*. Recent results using a reporter generated by genome engineering confirmed earlier findings that *egl-1* transcriptional induction occurs in all late pachytene cells upon treatment with IR, irrespective of whether germ cells undergo apoptosis or not (Hofmann *et al.* 2002; Doll *et al.* 2019). *egl-1* induction, therefore, correlates with the competency to undergo apoptosis as opposed to apoptosis induction *per se*. Indeed, germ cell apoptosis induction seems surprisingly complex, with mechanisms acting parallel to the CEP-1 pathway and BH3-only proteins (see below). Furthermore, germ cell apoptosis also involves cell nonautonomous mechanisms [for review, see Eroglu and Derry (2016)], and evidence for “bystander effects,” such that the level of DNA damage-induced apoptosis is modulated by secreted proteins or volatile compounds derived from animals pretreated with UV or IR (Peng *et al.* 2017; Tang *et al.* 2020).

Analysis of strains defective for the SIR-2.1 histone deacetylase, orthologous to human SIRT1, provided the first example of a mutant largely defective for DNA damage-induced apoptosis, but with normal ATM/ATR checkpoint-dependent *egl-1* and *ced-13* induction (Greiss *et al.* 2008b). It is currently not known how SIR-2.1 acts mechanistically. SIR-2.1 translocates from the nucleus to the cytoplasm during early apoptosis, at a stage where the nuclear envelope is still intact. SIR-2.1 translocation correlates with CED-4 accumulation at the nuclear periphery, where SIR-2.1 has the potential to transiently interact with, and regulate, CED-4 function. The HECT-domain E3 ligase EEL-1, which is homologous to human Huwe1/ARF-BP1/Mule, was shown to genetically behave like SIR-2.1 (Ross *et al.* 2011), as does KRI-1, the ortholog of human *KRIT1*/*CCM1*, a gene frequently mutated in the neurovascular disease cerebral cavernous malformation (Ito *et al.* 2010) (see below). Another recent study reported that IR-induced apoptosis is compromised in *ulp-3* mutants, which, like *sir-2.1* and *kri-1* mutants, are competent for checkpoint-dependent *egl-1* and *ced-13* induction (Bailly *et al.* 2019). ULP-3 is a protease needed to process branched poly-NEDD8 (ubiquitin-like) chains. Using *C. elegans* and human cells, the authors demonstrate that branched poly-NEDD-8 chains are able to bind to and inhibit the HSP-70/HSP70 chaperone. Given that HSP70 is required for CED-4/Apaf-1 oligomerization, HSP70 inhibition blocks apoptosis induction. Indeed, monomeric NEDD-8 appears to directly activate HSP70 and thus promote apoptosis (Bailly *et al.* 2019) (Figure  6).

Further evidence suggests that some DDRs leading to germ cell apoptosis are not mediated by the ATR and ATM sensor kinases. For instance, a deletion mutation that specifically affects the longest isoform of the sole *C. elegans* RAD-51 recombinase allows for the recombinational repair of SPO-11-induced breaks, while leading to a defect in DNA damage-induced apoptosis (Germoglio and Adamo 2018). Apoptosis is also defective in the absence of conserved HJ resolving enzyme GEN-1 (Bailly *et al.* 2010), in *msh-4* and *msh-5* mutants (Silva *et al.* 2013), and in *top-3* (Topoisomerase 3) mutants (Janisiw *et al.* 2018; Dello Stritto *et al.* 2021). In all these mutants, checkpoint signaling, as measured by *egl-1* transcriptional induction, is fully intact. Interestingly, the disassembly of RAD-51 upon IR treatment is delayed in all of these mutants, suggesting that delayed RAD-51 filament disassembly may lead to a block in apoptosis induction (Ackermann *et al.* 2016). Indeed, mutants defective for the UDF-2 E4-ubiquitin ligase, which is proposed to act by promoting RAD-51 filament disassembly in conjunction with the CDC-48 ubiquitin segregase, show a similar defect in apoptosis induction (Ackermann *et al.* 2016). The blockage of IR-induced apoptosis in *top-3* mutants likely involves a late recombination intermediate as apoptosis is reinstalled upon RAD-51 depletion (Dello Stritto *et al.* 2021). Interestingly, the *top-3* radiation-induced apoptosis defect is bypassed in mutants defective for NHEJ and MMEJ (Dello Stritto *et al.* 2021). All in all, distinct intermediates of recombinational repair appear to be able to block or activate DNA damage-induced apoptosis (Figure  6).

The link between DNA damage processing, HR and DNA damage checkpoint signaling is also illustrated by studies on UV-induced germ cell apoptosis. This, like the checkpoint acting in response to IR, requires CEP-1 and the core checkpoint and apoptosis pathways (Stergiou *et al.* 2007). Intriguingly, checkpoint activation also requires the XPF-1 and XPG-1 endonucleases, which are required for excising the damaged strand by causing an incision 5′ and 3′ of the UV-induced lesion (Stergiou *et al.* 2007). If UV-induced lesions are in close proximity and on opposite strands, these nucleases produce DSBs, which when subjected to further processing, trigger the DNA damage checkpoint. Consistent with DSB processing being an integral part of checkpoint signaling, strains mutant for the MRE-11 and RAD-54 recombination enzymes are also defective for checkpoint-induced apoptosis (Stergiou *et al.* 2011).

A report of a role of ceramide in germline apoptosis provides another example of apoptosis induction not linked to the canonical ATR/ATM checkpoint pathways (Deng *et al.* 2008). Ceramide is a central molecule in sphingolipid metabolism required for plasma membrane integrity, and in some reports, ceramide levels increase after treatment with apoptotic stimuli, such as exposure to UV and IR, or treatment with Tumor Necrosis Factor. It was proposed that ceramides form channels in the outer mitochondrial membrane, thereby releasing proapoptotic factors. In *C. elegans*, ceramide accumulates upon apoptosis induction, and mutants defective for ceramide biogenesis completely abrogate IR-induced apoptosis, a phenotype bypassed by microinjecting long-chain ceramides into the worm gonad (Deng *et al.* 2008). Interestingly, ceramide synthesis-defective mutants do not affect *egl-1* and *ced-13* transcriptional induction (Deng *et al.* 2008), and like SIR-2.1 and the aforementioned recombination genes, do not affect developmental and physiological germ cell apoptosis. This form of apoptosis does not require CEP-1 and is thought to maintain germ cell homeostasis. Recent evidence suggests that ceramide-dependent apoptosis requires PMK-1 and MPK-1 MAP kinase pathways (Yang *et al.* 2021) (Figure  6).

### Cell nonautonomous checkpoint signaling pathways

While studies on DDRs typically focus on cell autonomous mechanisms, the *C. elegans* system has uncovered cases where somatic cells modulate checkpoint pathways cell nonautonomously in the germ line (Sendoel *et al.* 2010). For example, amphid neurons sense oxygen levels and block IR-induced germ cell apoptosis under hypoxia conditions. Signaling is mediated by the conserved HIF-1 transcription factor, which acts as a sensor of low oxygen pressure in amphid sensory neurons and mediates the expression of the TYR-2 tyrosinase. TYR-2 is secreted from amphid sensory neurons and taken up by the gonad, suggesting that TYR-2, or a product of TYR-2 activity, is able to block IR-induced apoptosis. Why hypoxia leads to a blockage of DNA damage-induced apoptosis remains mysterious.

A recent study provides a mechanistic explanation for how KRI-1, a scaffolding protein expressed in somatic tissues, blocks DNA damage-induced apoptosis induction in the germ line (Chapman *et al.* 2019). As mentioned above, MAP kinase signaling in the pachytene region of the germ line is required for apoptosis induction (Rutkowski *et al.* 2011), and such MAP kinase signaling is blocked in *kri-1* mutants (Chapman *et al.* 2019). The reason for this is surprising and involves excessive levels of Zn2^+^ ions (Chapman *et al.* 2019), which are known to inhibit MAP kinase signaling likely at the level of the KSR-1 scaffold protein or the RAF-1 kinase (Jirakulaporn and Muslin 2004; Yoder *et al.* 2004). Storage of Zn2^+^ in gut granules is compromised in *kri-1* mutants, and this leads to excessive Zn2^+^ throughout the animal (Chapman *et al.* 2019). KRI-1 is required to maintain the activity of the KLF-3 transcription factor, which in turn, through regulating Zn2^+^ transporters, is required for restricting Zn2^+^ to gut granules. KRI-1 acts *via* the conserved adaptors ICAP-1 and CCM-2, which, like KRI-1, are linked to cerebral cavernous malformation by curtailing the activity of the ERK-5 MAP kinase pathway, the overactivation of which inhibits KLF-3 (Chapman *et al.* 2019). The role of KRI-1 and vertebrate orthologs KRIT1/CCM1 in regulating Zn homeostasis is conserved and likely relevant for understanding the underlying human disease (Chapman *et al.* 2019) (Figure  6).

CEP-1, besides mediating IR-induced germ cell apoptosis and UV-induced cell cycle arrest in adult gonads (Derry *et al.* 2007), also functions in the germline primordium of early L1 stage larvae. CEP-1 acts in the two germ cell precursor cells, Z2 and Z3, to mediate cell cycle arrest upon exposure to UV or IR (Ou *et al.* 2019). In a genetic screen for additional factors required for this response, the specialized eIF4E2 translation initiation factor, IFE-4, was identified and shown to mediate CEP-1 induction. Surprisingly, IFE-4 acts in the two somatic cells of the germ line primordium, Z1 and Z4, and is upregulated by UV irradiation. Damage signaling between the two somatic cells and the Z2 and Z3 germ cells in the primordium appears to be complex and involves Fibroblast Growth Factor signaling. Both the EGL-15 EGF receptor and the EGL-17 EGF growth factor are required for the induction of IFE-4 in somatic cells. EGF signaling likely involves an amplification loop and autocrine and paracrine circuits occurring in germ cells as well as the surrounding somatic cells of the gonad. Another receptor tyrosine kinase pathway, the FGRF-related SERF, is required to receive the stress signal in the germ cell niche to trigger CEP-1 expression and checkpoint-induced cell cycle arrest. Z2 and Z3 can be considered stem cells embedded in a stem cell niche composed of somatic cells. Indeed, evidence for the same mode of nonautonomous DNA damage signaling was observed in mammals: the hair follicle stem cell niche is composed of stem cells surrounded by nondividing support cells. Intriguingly, UV-induced p53 expression in stem cells requires IFE4 induction in somatic cells, demonstrating that UV-induced p53 signaling might also be noncell-autonomous in mammalian systems (Ou *et al.* 2019). Thus, *C. elegans* genetics allowed for uncovering multiple cell nonautonomous mechanisms of DNA damage signaling.

### Links between DNA damage signaling and organismal stress responses

Using *C. elegans* as an organismal model system has allowed for investigating links between DNA damage sensing and organismal stress response. Freely citing the German philosopher Nitsche, “anything that does not kill you makes you stronger”; in other words, overcoming a problem may generally make an organism more resilient. Indeed, treating *C. elegans* with UV, IR, or HU leads to an increased resistance to heat shock and oxidative stress (Ermolaeva *et al.* 2013). DSBs are likely to trigger the increased stress resistance, as strains where excessive meiotic DSBs accumulate due to defects in chromosome pairing become stress resistant (Ermolaeva *et al.* 2013). The signal that mediates such organismal response requires germline DNA damage and involves MAP kinase signaling (Greiss *et al.* 2008a; Kimura *et al.* 2012; Ermolaeva *et al.* 2013). Interestingly, the transcriptional response to IR, with the notable exception of the CEP-1-dependent *egl-1* and *ced-13* proapoptotic genes, is not dependent on the canonical DNA damage checkpoint signaling pathway (Greiss *et al.* 2008a). Many genes upregulated in response to IR are also upregulated in longevity mutants, suggesting that IR and aging induce an overlapping stress response program. Surprisingly, the IR transcriptional response also overlaps with the response triggered by pathogenic bacteria (Greiss *et al.* 2008a; Kimura *et al.* 2012; Ermolaeva *et al.* 2013); low dose IR protects against bacterial infection (Greiss *et al.* 2008a; Kimura *et al.* 2012; Ermolaeva *et al.* 2013). The most notable IR-induced gene is the *mul-1* mucin (Kimura *et al.* 2012). Mucins are highly glycosylated secreted proteins that form protective gel-like structures, helping to maintain mucosal barriers. IR-dependent *mul-1* induction requires the ELT-1 and DAF-16 transcription factors, and p38 MAP kinase signaling. *mul-1* depleted L1 larvae are hypersensitive to IR, consistent with a protective role for mucin in response to damage.

How exactly IR and DNA damage are interlinked with organismal stress response remains to be understood, but it is known to involve the ubiquitin-proteasome system and enhanced proteostasis (Ermolaeva *et al.* 2013). Organismal signaling in response to IR appears to be widespread: Microbeam irradiation of the pharynx or rectal regions of the worm also leads to elevated germ cell apoptosis. Such germ cell apoptosis induction appears to involve germ cell DNA damage, and is mediated by MAP kinase signaling (Guo *et al.* 2013). In line with hormetic, organismal signaling, microbeam irradiation restricted to small parts of the body renders worms partially refractory to subsequent apoptosis induction following exposure to IR (Tang *et al.* 2016). In conclusion, DNA damage repair and damage response seems to be interconnected with the organismal stress response.

## Using *C. elegans* to define mutational signatures

The *C. elegans* life cycle provides an ideal experimental system to investigate the mutagenic processes that result from the combination of primary DNA lesions inflicted by DNA damaging agents or DNA replication failure, and the DNA repair machinery [for reviews, see Meier and Gartner (2014) and Meier *et al.* (2020)]. Given the hermaphroditic nature of *C. elegans* reproduction, mutagen exposure is applied such that germ cells are exposed and gametes derived from these cells fuse to form the zygote. Development into self-fertilizing adults allows for the clonal amplification of any mutations that are fixed before the first zygotic division; consequently, genomic DNA is conveniently prepared from the immediate progeny of the original F1 animal. Mutations in the animals derived from the first F1 are expected to occur at a frequency of close to 50%, in line with mutations being heterozygous, allowing for accumulation of a massive load of heterozygous mutations or complex rearrangements and their analysis by next generation sequencing. Indeed, the hermaphroditic life cycle allows for studying mutagenesis even without exposure to DNA damaging agents. Propagation of three to five parallel lines for 20–40 generations is necessary to establish mutation rates in wild type (Cheung *et al.* 2002; Denver *et al.* 2006; Lipinski *et al.* 2011; Meier *et al.* 2014, 2018; Volkova *et al.* 2020).

Mutational spectra encompass information about all possible single nucleotide variants (SNVs), C > A, C > G, C > T, T > A, T > C, and T > G, and their distribution in the sequence context. In addition, spectra include information about dinucleotide mutations and the composition of indels—small insertion, deletions, or combined insertion/deletions—stratified based on their size. Finally, spectra also reveal SVs, larger (>1,000 bp) events grouped into deletions, inversions, tandem duplications, and translocations (Alexandrov *et al.* 2013). Comparing mutational signatures between wild-type and DNA repair defective worms allows for assessing the contribution of various repair pathways in mending DNA lesions that occur during normal *C. elegans* proliferation, or as a consequence of exposure to genotoxic agents. Distinct mutagenic scars provide mechanistic insight into mutagenic processes (Cheung *et al.* 2002) (see below). Importantly, experimentally-derived mutational signatures are often conserved. Once the differential nucleotide composition of human and *C. elegans* genomes is taken into consideration, *C. elegans* signatures can be compared to those derived from the analyses of thousands of cancer genomes, helping to decipher the primary mutagenic causes that trigger oncogenic transformation (Meier *et al.* 2014, 2018; Volkova *et al.* 2020).

### Mutational spectra accumulating in unchallenged wild type, HR, and MMR mutants

Under unchallenged conditions, wild type and many DNA repair defective strains show low mutation rates of around 0.8–2 mutations per generation. Assuming that 15 cell divisions are needed to pass the germ line from one generation to the next, the mutation rate per nucleotide per cell division is ∼6.7× 10^−10^ in wild type, which compares well to estimates of ∼0.45 × 10^−10^ per nucleotide per division for the human male germ cell lineage (Denver *et al.* 2009; Kong *et al.* 2012; Meier *et al.* 2014, 2018, 2021; Meier and Gartner 2014; Konrad *et al.* 2019).

In general, about half of the ∼40 DNA repair defective strains available show a two- to fivefold increase in background mutagenesis (Volkova *et al.* 2020; Meier *et al.* 2020). Notably, no increase in mutagenesis is observed in the apoptosis-defective CEP-1/p53 mutant, in strains defective for the FA pathway, or DNA end-joining mutants. Mutagenesis is increased by approximately twofold in NER mutants. Mutation of the uracil-DNA-glycosylase (UNG-1) leads to increased C > T changes, potentially generated by uracil–adenine pairing, consistent with UNG-1 eliminating uracil arising from misincorporation or spontaneous cytosine deamination (Meier *et al.* 2014; Volkova *et al.* 2020). Mutational signatures associated with defective HR can be grouped into two classes (Meier *et al.* 2021): one, mutants lacking BRC-1, RAD-51, or RAD51 paralogs show elevated base substitutions, indels, and SVs, features observed in *brca1*-defective cancer genomes and in mammalian mutation accumulation lines. Two, HR-defective *mus-81* and *slx-1* nuclease mutants, as well as *him-6*, *helq-1*, and *rtel-1* helicase mutants, primarily accumulate SVs. *helq-1* mutants accumulate tandem repeats, where breakpoints are associated with inverted repeat sequences, suggesting a specific role for HELQ-1 in reading through stem loop structures. A unique pattern of “translocation” events involving homeologous sequences occurs in *rip-1* (RAD51 paralog) mutants, indicative of aborted strand invasion events (Meier *et al.* 2021). Finally, while inactivation of *cep-1* does not affect mutagenesis, the combined deficiency of *brc-1* and *cep-1* display increased, locally clustered mutagenesis compared to *brc-1* mutants alone, suggesting that checkpoint signaling is important when DNA repair is abrogated (Meier *et al.* 2021).

*C. elegans mlh-1* and *pms-2* MutL MMR mutants lead to the highest level of mutations of any DNA repair background analyzed, ∼60 per generation, with ∼1/3 being base substitutions, and the remainder being small insertion and deletions enriched in homopolymer repeat sequences (Degtyareva *et al.* 2002; Tijsterman *et al.* 2002; Denver *et al.* 2005; Meier *et al.* 2018). The SNV signature associated with MMR deficiency consists of a characteristic set of C > A, C > T, and T > C mutations, likely caused by DNA replication failure (Meier *et al.* 2018). The *C. elegans* MMR signature helped to confirm that the computationally deduced cancer signature COSMIC 20 (Catalogue of Somatic Mutations in Cancer) (Alexandrov *et al.* 2013, 2020) is directly related to MMR deficiency (Meier *et al.* 2018). Indeed, based on the worm MMR signature, *de novo* signature extraction from 215 human colorectal and 289 gastric adenocarcinomas allowed for uncovering a conserved MMR signature in ∼20% of those tumors, 98% of which showed microsatellite instability (Meier *et al.* 2018). Excessive mutagenesis in MMR cancers is associated with the formation of neoantigens that sensitize those cancers for cancer immunotherapy (Schwitalle *et al.* 2008; Le *et al.* 2017; Mardis 2019). It is surprising that microsatellite testing is not more commonly used to stratify treatment of gastrointestinal cancers, and possibly cancers where MMR defects are common, to identify tumors across tissue types that would respond to immunotherapy (Meier *et al.* 2020).

### Mutational processes associated with DOG-1/FANCJ and TLS polymerase deficiencies

*dog-1* (FANCJ helicase) was identified in a seminal study as a locus that, when mutated, causes increased rates of mutagenesis at G-rich sequences (Cheung *et al.* 2002). Such sequences have the potential to form G4 structures, where guanines stack into stable, four-stranded non-Watson-Crick tertiary DNA structures that impede replication fork progression (Cheung *et al.* 2002; Tarailo-Graovac *et al.* 2015). Deletions associated with G-rich DNA in *dog-1* mutants have a surprisingly uniform size, ranging from 50 to 300 base pairs (Koole *et al.* 2014). Careful analysis of the deletion breakpoints revealed that these are commonly flanked by sequences that show microhomology, a genomic scar the authors postulated to be associated with MMEJ activity. Indeed, this was confirmed by an increased deletion size and no microhomology at breakpoints in *dog-1; polq-1* (polymerase theta) double mutants (Koole *et al.* 2014; Roerink *et al.* 2014). Both *in vivo* and *in vitro* experiments suggest that POLQ-1 stabilizes structures where resected 3′ single-stranded overhangs pair at their complementary terminal nucleotide(s) to prime DNA synthesis (Wood and Doublié 2016; Seol *et al.* 2018; Schimmel *et al.* 2019; Brambati *et al.* 2020). POLQ-1-dependent MMEJ is a major *C. elegans* repair modality, with POLQ-1 also mending DSBs, and deletions caused by *rev-1* and *rev-3* (polymerase zeta) translesion polymerase mutations (Koole *et al.* 2014; Roerink *et al.* 2014; van Bostelen *et al.* 2020), by *polh-1, polk-1* double mutants (van Bostelen *et al.* 2020), by treatment with EMS (van Schendel *et al.* 2016), by UV–TMP treatment (see below) (van Schendel *et al.* 2016), as well as by HR deficiency (van Bostelen *et al.* 2020).

### Chromosome fusions resulting from critically short telomeres

When telomeres become critically short due to the absence of telomerase activity, chromosome end-to-end fusions arise. These were observed in *C. elegans* strains defective for telomerase, after propagation over >10 generations, and occur concomitant with the onset of reduced fecundity before clonal lines become sterile (Ahmed and Hodgkin 2000; Meier *et al.* 2006). Using array technology and genome sequencing, fusions were linked to copy number changes, likely involving replication fork stalling and template switching that result in replication-induced duplication processes close to the fusion sites (Lowden *et al.* 2011). Other chromosome fusions showed scars indicative of repeated chromatid breakage-fusion-bridge cycles, with a final interchromosomal event related to chromothripsis, a mutagenic process that involves the localized random integration of broken chromosome fragments into the fusion site (Stephens *et al.* 2011; Meier *et al.* 2014). The same pattern of chromosome fusions is observed in lymphoblastic leukemia (Li *et al.* 2014). Telomere-proximal complex SVs, possibly involving chromosome-to-chromosome-to-chromosome fusions, are also observed in *atm-1* mutants, likely because some telomeres are critically short in this background, albeit telomere attrition-linked progressive sterility is not found (Jones *et al.* 2012; Meier *et al.* 2021). Finally, genetic suppressors that bypass telomere shortening-induced sterility were isolated. These suppressor lines carry translocations or amplifications of genomic regions, termed TALT1 and TALT3 at subtelomeric regions (Seo *et al.* 2015; Kim *et al.* 2019). It is assumed that these sequences serve as templates for a recombinogenic, telomerase-independent alternative mode of telomere lengthening (ALT) mechanism. ALT is commonly used in cancer cells.

### Mutational signatures associated with mutagen exposure

A wide variety of mutagens have been used to study DNA damage and have well-characterized mutational signatures. EMS, N-ethyl-N-nitrosourea (ENU), and UV–TMP are most commonly used in *C. elegans* mutagenesis screens, and were the first genotoxins where mutational signatures were defined. EMS causes G > C to A > T transitions, ENU causes a flat SNV signature with a modest preference for G > C to A > T changes, and UV–TMP exposure leads to base substitutions affecting all bases equally in addition to small deletions averaging 400–500 bases; such deletions also occur upon EMS and ENU exposure, albeit at a much lower frequency (Greenwald and Horvitz 1980; De Stasio and Dorman 2001; Flibotte *et al.* 2010). These deletions likely arise when alkylated bases fail to be repaired or bypassed; the 5′ breakpoint occurs right after a damaged cytosine in the case of EMS, and adenine in the case of UV–TMP treatment (van Schendel *et al.* 2016; Schimmel *et al.* 2019). Breaks appear to be processed by MMEJ, as characteristic scars of 400–500 bases deletions with flanking microhomology are observed. The methylating agents MMS and DMS produce mutation spectra with predominating T > A and T > C substitutions (Volkova *et al.* 2020). Exposure to aristolochic acid and aflatoxin B1, both of which form bulky-adducts, leads to spectra where C > A and T > A substitutions predominate, as seen in human spectra exposed to these agents (Volkova *et al.* 2020). UV light exposure leads to C > T transitions in a C/TCA/C/T context, similar to the signature associated with exposing human cells to simulated UV light, and COSMIC cancer signature 7a+b linked to UV-induced melanoma (Volkova *et al.* 2020). In contrast, IR leads to a flat spectrum where changes in all bases are equally likely, together with indels and SVs (Volkova *et al.* 2020). Exposure to cisplatin, a widely used anticancer chemotherapeutic agent that causes DNA monoadducts, inter- and intrastrand crosslinks, largely leads to C > A transversions enriched in a CCC and CCG context in addition to SVs (Meier *et al.* 2014; Volkova *et al.* 2020). While monoadducts and intrastrand crosslinks are the most common modifications caused by cisplatin, ICL, which are rare but may cause dramatic mutagenic outcomes, are likely the most cytotoxic event (Meier *et al.* 2014). Indeed, exposure to cisplatin and mechlorethamine (nitrogen mustard), another DNA crosslinking agent, leads to rare cases of complex, localized, large-scale genomic rearrangements. This signature is similar to chromoanasynthesis (“chromo” for chromosomes and “anasynthesis” for reconstitution), first described in inherited constitutional genomic disorders, which involves localized copy number changes likely originating from impeded DNA replication and fork collapses that lead to a series of microhomology-driven invasions into nearby genomic regions (Liu *et al.* 2011). Such rearrangements triggered by several chemically distinct DNA crosslinking agents are consistent with persistent DNA crosslinks being the cause for chromoanasynthesis (Meier *et al.* 2014; Tam *et al.* 2015). In summary, multiple conserved mutational signatures associated with genotoxic agents were uncovered in *C. elegans*, many of which have provided insight into human mutational signatures.

### The interplay between primary DNA damage and DNA repair pathways shapes genotoxin-induced mutational signatures

The *C. elegans* system has been critical in uncovering how DNA repair factors prevent mutagenesis caused by the exposure to various genotoxic agents. A recent, systematic analysis probing the effect of 11 mutagens on a panel of *C. elegans* wild type and DNA repair-defective mutants revealed that in approximately 40% of all tested cases repair defects were associated with increased mutagenesis, a change in the mutation spectrum, or a combination of both (Volkova *et al.* 2020). Up to 98% of SNVs induced by EMS, MMS, and DMS are prevented by POLK-1, while NER mends ∼97% of UV-induced lesions. In contrast, *rev-3*, and to some extent *polh-1* translesion polymerase mutants, showed reduced SNVs upon UV treatment or exposure to EMS, MMS, aflatoxin, and aristolochic acid (Volkova *et al.* 2020), in line with earlier reports on translesion polymerases (Lawrence and Hinkle 1996; Li *et al.* 2002; Diaz *et al.* 2003; Yoon *et al.* 2019). The reduction of SNVs, however, comes at the expense of an increased burden of SVs. Thus, being defective for the error prone bypass of modified bases, leads to an increased number of indels and SVs, which tend to be biologically more harmful.

Careful analysis of DMS, MMS, and EMS-induced signatures provide an example of the extreme redundancy of DNA repair pathways: MMS and DMS (besides methylating adenine and guanine at the N7 position that do not affect base pairing) lead to the formation of O6-methylguanine which results in C > T changes, and N3-methyladenine which causes T > A and T > C changes (Beranek 1990; Volkova *et al.* 2020). EMS mostly induces O6-ethylguanine adducts, with a small proportion of N3-ethyladenine (Brookes and Lawley 1961). AGT-1, the *C. elegans* homolog of O6-methylguanine DNA transferase, appears to remove the O6-methyl group from guanine, thus preventing C > T substitutions, but does not contribute to the repair of N3-methyladenine (Volkova *et al.* 2020). Translesion polymerase kappa (POLK-1) bypasses N3-methyladenine in an error-free way, in line with massively increased T > A and T > C changes in *polk-1* mutants (Volkova *et al.* 2020). A 10-fold increase of T > A/C changes in EMS treated *polk-1* mutants indicates that this translesion polymerase is also capable of bypassing N3-ethyladenine in an error-free way. The aforementioned reduction of MMS-induced SNVs in *rev-3* mutants is indicative of the error-prone bypass of N3-methyladenine, which prevents the formation of indels and SVs. In contrast, REV-3 does not have a major role in DNA repair upon EMS treatment. Finally, NER defective mutants show a twofold increase of MMS, DMS, and EMS-induced mutations, indicating that NER is able to excise damaged sequences that contain O6-methyl and ethylguanine as well as N3-methyl- and ethyladenine, albeit to a lesser extend compared to bulky aristolochic acid adducts (Volkova *et al.* 2020).

In summary, multiple DNA repair pathways mend DNA lesions. It is likely that the relative contributions of DNA repair pathways vary between organisms and between cell types of the same organism. This is conceptually in line with tumors linked to DNA repair defects, which are often surprisingly tissue-specific. For example, inherited defects for HR predispose individuals to breast and ovarian cancer, while MMR deficiency is linked to gastrointestinal and uterine cancers. The analyses of mutation profiles in *C. elegans* provide insights as to how primary DNA lesions are mended by DNA repair, with important implications for understanding carcinogenesis.
